# Curcumin-Based Nanomedicines in the Treatment of Inflammatory and Immunomodulated Diseases: An Evidence-Based Comprehensive Review

**DOI:** 10.3390/pharmaceutics15010229

**Published:** 2023-01-10

**Authors:** Lucas Fornari Laurindo, Gabriel Magno de Carvalho, Bárbara de Oliveira Zanuso, Maria Eduardo Figueira, Rosa Direito, Ricardo de Alvares Goulart, Daiene Santos Buglio, Sandra Maria Barbalho

**Affiliations:** 1 Department of Biochemistry and Pharmacology, School of Medicine, University of Marília (UNIMAR), Avenida Hygino Muzzy Filho, 1001, Marília, São Paulo 17525-902, Brazil; 2 Laboratory of Systems Integration Pharmacology, Clinical & Regulatory Science, Research Institute for Medicines (iMed.ULisboa), Faculdade de Farmácia, Universidade de Lisboa, Av. Prof. Gama Pinto, 1649-003 Lisboa, Portugal; 3 Faculty of Pharmacy, Universidade de Lisboa, Av. Prof. Gama Pinto, 1649-003 Lisboa, Portugal; 4 Postgraduate Program in Structural and Functional Interactions in Rehabilitation, University of Marília (UNIMAR), Avenida Hygino Muzzy Filho, 1001, Marília, São Paulo 17525-902, Brazil; 5 Department of Biochemistry and Nutrition, School of Food and Technology of Marília (FATEC), Avenida Castro Alves, 62, Marília, São Paulo 17500-000, Brazil

**Keywords:** curcumin, delivery systems, nanomedicines, curcumin-based nanomedicines, inflammation, autoimmunity, inflammatory diseases, auto-immune diseases

## Abstract

Curcumin (CUR) is a polyphenol extracted from the rhizome of *Curcuma longa* that possesses potent anti-inflammatory and antioxidant potential. Despite CUR’s numerous beneficial effects on human health, it has limitations, such as poor absorption. Nano-based drug delivery systems have recently been applied to improve CUR’s solubility and bioavailability and potentialize its health effects. This review investigated the effects of different CUR-based nanomedicines on inflammatory and immunomodulated diseases. PUBMED, EMBASE, COCHRANE, and GOOGLE SCHOLAR databases were searched, and the Scale for Assessment of Narrative Review Articles (SANRA) was used for quality assessment and PRISMA guidelines. Overall, 66 studies were included comprising atherosclerosis, rheumatoid arthritis (RA), Alzheimer’s disease (AD), Parkinson’s disease (PD), multiple sclerosis (MS), Huntington’s disease (HD), inflammatory bowel diseases (IBD), psoriasis, liver fibrosis, epilepsy, and COVID-19. The available scientific studies show that there are many known nanoformulations with curcumin. They can be found in nanosuspensions, nanoparticles, nanoemulsions, solid lipid particles, nanocapsules, nanospheres, and liposomes. These formulations can improve CUR bioavailability and can effectively be used as adjuvants in several inflammatory and immune-mediated diseases such as atheroma plaque formation, RA, dementia, AD, PD, MS, IBD, psoriasis, epilepsy, COVID-19, and can be used as potent anti-fibrotic adjuvants in fibrotic liver disease.

## 1. Introduction

The increase in chronic inflammatory and auto-immune diseases is alarming worldwide, and the search for new therapeutic approaches is necessary [1,2]. In this scenario, naturally occurring bioactive compounds have been considered as adjuvants in therapeutical practice since they are usually low-cost, have few collateral effects, and are simple to use. For example, since ancient times, turmeric (*Curcuma longa*) has been used to treat inflammatory and auto-immune conditions. Curcumin (CUR) is its primary phenolic bioactive compound and exhibits promising anti-inflammatory and auto-immune preventive potential in clinical and pre-clinical human studies [3,4].

Although *C. longa* possesses two other important curcuminoids (CURoids) (desmethoxycurcumin and bis-desmethoxycurcumin), CUR is considered the most important secondary metabolite. It is also known as (1E,6E)-1,7-bis(4-hydroxy-methoxyphenyl)-1,6-heptadiene-3,5-dione, consisting of two aromatic structures containing methoxy and hydroxy groups linked via a seven-carbon-containing chain of a moiety of α,β-unsaturated β-diketone [1,5,6].

Besides the potent anti-inflammatory and auto-immune protective effects of CUR, it shows hydrophobicity, poor solubility, rapid clearance, and low stability, which limits its usability in daily clinical practice. CUR-based nanomedicines could improve solubility, absorption, clearance, and stability [7,8]. Among different types of nanocarriers, biologically derived and other biopolymer carriers have attracted scientific attention due to their pleiotropic features [9]. Currently, therapeutic CUR-based nanomedicines have been used mainly against inflammatory cardiovascular diseases [10]. However, in recent years, bioengineers and other researchers started applying CUR nano-formulations to treat other conditions with inflammatory and auto-immune characteristics. This review aims to summarize the studies using nanomedicines as new delivery systems of CUR-based therapies for treating inflammatory and auto-immune diseases.

## 2. Inflammatory Process: An Overview

The inflammatory response represents one of the most critical components of the execution of innate and adaptive immune responses. Although it is part of the defense mechanisms against numerous aggressions, in many cases, it can also cause damage [11,12]. Inflammation is a refined biological process controlled by the balance between the action of pro- and anti-inflammatory substances. The disharmony and prolonged exposure to pro-inflammatory cytokines, such as Interleukin (IL) -1β, IL-6, Tumor factor necrosis α (TNF-α), and Interferon (IFN) -γ can lead to the development of chronic inflammation [11,13]. Mainly, this process is triggered by the inefficiency of anti-inflammatory mechanisms, which can allow immune self-aggression to body tissues [11,14].

The inflammatory process begins after identifying the pathogen-associated molecular pattern (PAMP), a biological stressor, or after the release of substances derived from cell lysis. Epithelial cells react to these substances releasing cytokines (TNF-α, IL-1, and IL-18), which are responsible for activating the vascular endothelium and promoting leukocyte migration [11,15,16].

With exudative events, the increased leukocyte load at the site of inflammation increases the production of pro-inflammatory compounds. However, these same leukocytes are responsible for releasing anti-inflammatory mediators, which decrease the peripheral effects of local inflammatory sites and reduce the damage caused by pro-inflammatory drugs. Anti-inflammatory mediators are primarily composed of growth factors that also have anti-inflammatory action, such as transforming growth factor-β (TGF-β), vascular endothelial growth factor (VEGF), and fibroblast growth factor (FGF) [11,17,18]. Immune cells can also alter pro-inflammatory processes by producing reactive oxygen species (ROS) [11].

Regenerative and healing processes depend on the stimulus generated by substances released by leukocytes (M2 macrophages), such as IL-13 and TGF-β, which activate the proliferation of fibroblasts and, especially, VEGF to stimulate angiogenesis. However, some cells cannot multiply; therefore, the injured tissue is replaced by connective scar tissue. In healing, growth factors such as FGF-α and β, TGF-β and platelet-derived growth factor (PDGF), which stimulate the synthesis of matrix compounds, as well as their deposition at the injured site, stand out in importance [11,19,20,21].

## 3. CUR, a Phenolic Compound Derived from *Curcuma longa*

*C. longa* is a plant from India cultivated in different parts of the world, mainly in Southeast Asia. It has been used as a seasoning for curry preparation due to its color and flavor. In addition to this traditional culinary use, the plant can also be used as a natural dye, insect repellent, and for medicinal purposes. The latter has gained prominence today and has acted as an object of study in several laboratories, given the therapeutic potential of turmeric components against inflammatory, oxidative, neoplastic, and aging conditions. It comprises carbohydrates, proteins, lipids, water, minerals, vitamins, and CURoids [22]. CUR, desmethoxycurcumin (DMC), and bisdemethoxycurcumin (BMC) are the CURoids in their composition. Among them, CUR comprises more than 70% of the curcuminoid (CURoid) content and has been widely identified as primarily responsible for the benefits associated with the plant [23].

CUR is a phenolic compound that has two tautomeric forms, keto and enol. Due to its lipophilicity, it is not well absorbed in aqueous solutions and at room temperature, either at neutral or acidic pH, in which the keto tautomer is predominant. The enol tautomer is predominant under alkaline conditions, and under these conditions, CUR is better absorbed; however, it is degraded more quickly [24].

CUR promotes the downregulation of molecules and signaling pathways associated with inflammatory processes such as cyclooxygenase (COX) and lipoxygenase, inducible nitric oxide synthase (iNOS), soluble vascular cell adhesion molecule [sVCAM]-1, ILs, TNF-α, and Nuclear factor-κB (NF-κB) [3]. The different pharmacological responses associated with CUR are generated from the modulation of other biological targets such as transcription factors, growth factors, inflammatory cytokines, enzymes, cell cycle proteins, and different cell signaling pathways. NF-κB is downregulated by CUR via some other mechanisms. One example is the action on the peroxisome proliferator-activated receptor γ (PPARγ). At the expense of inhibiting NF-κB pathways, anti-inflammatory effects are produced, for example, by blocking the assembly and activation of the NOD-like domain-containing pyrin receptor 3 (NLRP3), a multiprotein complex associated with the genesis of many inflammatory diseases [25].

The inhibition of NF-κB also modifies the expression of inflammatory cytokines. Expressions of TNF-α, IL-1, IL-6, and IL-8 are suppressed, as is the expression of the protein kinase (PK), mammalian target rapamycin activity (mTOR), and mitogen-activated PK pathways (MAPKs). Another mechanism associated with decreased expression of inflammatory cytokines is the regulation of the activator protein-1 (AP-1), with a consequent reduction in the expression of genes that encode various pro-inflammatory cytokines [26,27]. On the other hand, CUR increases the expression of heme oxygenase-1 (HO-1), contributing to the switch from a pro-inflammatory to an anti-inflammatory environment by suppressing TNF-α and IL-1β and increasing IL-10 [28,29].

Oxidative stress (OS) is closely associated with the inflammatory scenario since the accumulation of ROS activates transcription factors responsible for the increase of inflammatory mediators. CUR also reduces OS by upregulating leucine zipper protein nuclear factor erythroid 2–related factor 2 (Nrf2), which modulates antioxidant properties, increasing levels of glutathione (GSH), superoxide dismutase (SOD), and catalase (CAT) enzymes, and decreasing malondialdehyde, thiols, and protein carbonyls [28,30]. CUR also has an action on nicotinamide adenine dinucleotide phosphate (NADPH) oxidase, contributing to a decrease in the proportion of ROS. In addition, the chemical structure of CUR favors its antioxidant activity since it donates hydrogen atoms from its phenolic groups [31].

Despite CUR’s numerous beneficial effects on human health, this compound has some important limitations that must be addressed, such as low water solubility (CUR is extremely hydrophobic), unstable chemical structure, rapid metabolization but poor absorption, as well as the fact its utilization and absorption depend on sex and species. The low solubility of CUR in aqueous solution and intestinal malabsorption impairs its bioavailability, limiting the range of its pharmacological effects [32]. However, recent clinical evidence has shown that chemicals modified by nanotechnology are proven to be highly effective as drug delivery systems and for targeting the required tissue throughout the human body. So, the obstacles that nature has imposed to the use of CUR clinically have been eliminated and truly overcome by the synthesis of CUR-based nanomedicines or CUR nanoparticles, liposomes, micelles, and phospholipid complexes that do not only deliver better CUR absorption and distribution to the target organs and tissues but also increase CUR circulation making it longer, permeability and resistance to the metabolic process [33,34,35]. Table 1 shows the potential therapeutic effects of CUR-based nanomedicines on some inflammatory and immunomodulated.

### 3.1. CUR-Based Nanomedicines in Atherosclerosis

A chronic inflammatory state characterizes atherosclerosis. Atheroma formation is a phenomenon that depends highly on modifying low-density lipoproteins (LDL) particles that emerge in the arterial vessels. The LDL particles are modified into atherogenic forms (oxidized LDL, desialylated LDL, and glycosylated LDL). Although atherosclerosis is a disease that can be implicated in severe damage in every large and medium-sized artery, atheroma plaques are commonly responsible for coronary artery disease and other cerebrovascular conditions that lead to ischemic heart and encephalic attacks [102,103].

**Figure 1:**
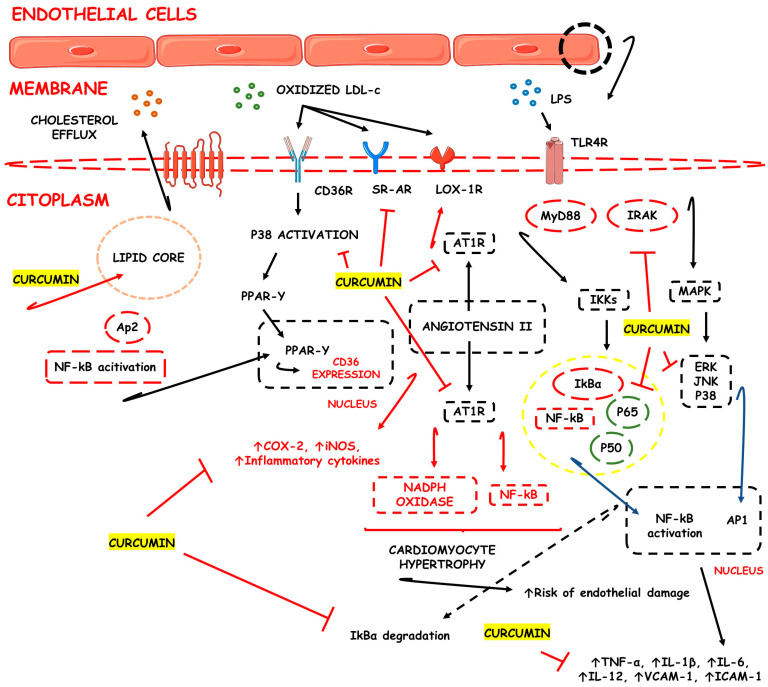
Possible mechanisms associated with CUR’s protective effects against atheroma formation. Atherosclerosis is a phenomenon that depends highly on modified LDL in the arterial vessels. CUR protects against atherosclerosis via anti-inflammatory and immunomodulating effects, as well as exerting antilipidemic actions and promoting MMP expression down-regulation. ↑, increase; Ap2, apetala type 2; AT1R; angiotensin 1 receptor; CD36R, CD36 receptor; COX-2, ciclooxigensase 2; ERK, extracellular signal-regulated kinases; ICAM-1, intercellular adhesion molecule 1; IkBa, nuclear factor of kappa light polypeptide gene enhancer in B-cells inhibitor alpha; IKKs, IκB kinases; IL, interleukin; IRAK, interleukin-1 receptor-associated kinases; JNK, N-terminal kinase; iNOS, inducible nitric oxide synthase; LDL-c, low-density lipoprotein cholesterol; LOX-1R, lysyl oxidase type 1; LPS, lipopolysaccharides; MAPK, mitogen-activated protein kinase; MyD88, myeloid differentiation primary response 88; NF-kB, nuclear factor kappa b; P38, p38 signaling transduction pathway; P50, NF-kB P50 heterodimer; P65, NF-kB P65 heterodimer; PPARγ, peroxisome proliferator-activated receptor γ; SR-AR, scavenger receptor type A; TLR4, tool-like receptor 4; TNF-α, tumor factor necrosis alfa; VCAM-1, vascular cell adhesion molecule 1.

Pathologically, lipid peroxidation of LDL seems to be the critical event to atherosclerosis occurrence. Oxidized LDL binds to β2-glycoprotein I to form circulating complexes that emerge into arteries and trigger inflammatory and immunogenic events that promote endothelial dysfunction and the synthesis and secretion of several different pro-inflammatory cytokines. These cytokines lead to inflammation and an accelerated auto-immune response that can promote the accumulation of lipids intracellularly within atherosclerosis plaques. The most important factor related to the inflammatory and auto-immune activation of the vessels is the β2-glycoprotein I and/or its complexes promoting early atherogenesis by stimulating the production of pro-inflammatory innate immunity firstly through endogenous sensors and inflammasome activation such as the interferon and IL-1 pathways [104,105]. Figure 1 shows the possible mechanisms associated with CUR’s protective effects against atheroma formation.

Jiang et al. [36] studied the *sonodynamic* therapy by stabilized polyvinylpyrrolidone (PVPK30) and sodium dodecyl sulfate (SDS) CUR nanosuspensions (CUR-ns) against atherosclerosis in vivo. The results showed that all forms of CUR-ns were more easily absorbed by the animals than the free Cur. Additionally, the results of this study showed that CUR-ns-SDS was more effective in reducing the total cholesterol levels of the animals and the LDL particles, and promoting the transformation from M1 to M2 macrophages, relieving atherosclerosis by inhibiting the progression of plaques by interfering with macrophages polarization. In the same study, the authors evaluated RAW264.7 cells and observed that CUR-ns-SDS enhanced macrophage apoptosis through mitochondrial interference.

Meng et al. [37] proposed a novel CUR-loaded nanoparticle to restrict atherosclerosis development and promote plaque stability in apolipoprotein E knockout mice. These authors evaluated the roles of a novel linear-dendrimer methoxy-poly (ethylene glycol)-b-poly(e-caprolactone) copolymer nanoparticle loading CUR (e-Cur-NPs) as anti-atherogenic. The results showed that e-Cur-NPs significantly decreased atheroma formation and atheroma lesions and were more effective in stabilizing vulnerable plaques compared to free CUR. As anti-atherosclerotic mechanisms, e-Cur-NPs include decreasing intraplaque microvessels concentration, inhibiting the metalloproteinase (MMP) -2 and MMP-9 activity, reducing inflammatory response and pro-inflammatory cytokines formation, and regulating LDL particle metabolism more effectively than free CUR. The distribution of CUR was also ameliorated by e-Cur-NPs encapsulation, which augmented this curcuminoid concentration in the thoracic aorta of the treated animals more preeminently compared with free CUR.

Li et al. [38] studied liposomes modified using a targeting ligand prepared to co-deliver atorvastatin and CUR to treat dysfunctional endothelial cells overexpressing E-selectin as a promising therapeutic technique against atherosclerosis occurrence and progression. Results showed that liposomes delivered with CUR and atorvastatin resulted in synergistic effects in suppressing the adhesion molecule expression and reducing the plasma lipid levels in the treated mice. Additionally, the authors addressed that the treatment could effectively reduce foam cell formation and the secretion of pro-inflammatory cytokines by blocking the monocyte migration into the *intima* layer of large to medium-sized arteries. Conclusively, CUR successfully reduced atorvastatin-induced endothelial cytotoxicity and prevented atherosclerosis.

Due to the above information, it can be concluded that CUR in the forms of polyvinylpyrrolidoneand SDS nanosuspensions, nanoparticles, and liposomes are effective against atheroma plaque formation. CUR-based nanomedicines were able to promote antilipidemic effects and decrease total serum cholesterol and LDL levels, limit macrophage inflammatory polarization augmenting M2 and reducing M1 macrophages and augment immune regulation of these cells, decrease atheroma progression and increase atheroma plaques stability, augment inflammatory macrophage apoptosis and diminish the activity of matrix MMPs (such as MMP-2) in the vessels and the intraplaque micro vessels concentration. CUR-based nanomedicines also limited pro-inflammatory cytokines production and release, adhesion molecules expression by the vessels, and monocyte migration into the intima layer of large to medium-sized arteries.

### 3.2. CUR-Based Nanomedicines in Rheumatoid Arthritis

Rheumatoid arthritis (RA) is triggered by a combination of genetic, epigenetic, and environmental factors that result in chronic destructive inflammation of the articular cartilage and extra-articular manifestations, which may involve the skin, heart, blood vessels, and lungs. The factors most associated with the genesis of RA, such as smoking and human leukocyte antigen (HLA) -DRB genes, seem to increase the expression of the enzyme peptidyl arginase deiminase (PADI) capable of converting arginine residues into citrulline, producing citrullinated peptides that act as neoepitopes and, thus, recruit and activate self-reactive immune cells, initiating an auto-immune inflammatory process [106,107,108]. This inflammatory process produces inflammatory cytokines such as TNF-α, IL-1, IL-6, MMPs, chemokines, vasoactive peptides, and oxygen and nitrogen intermediates by synovial macrophages. In addition, immune activation induces the production of anti-citrullinated peptide antibodies (ACPAs) and IgA and IgM antibodies that bind to the Fc portion of G immunoglobulins, called rheumatoid factors (RFs), which are present in most patients with AR [109,110].

Zheng et al. [39] investigated the effects of CUR on adjuvant-induced RA (AIA) in Sprague Dawley rats. The animals were randomly divided into groups treated with CUR in intravenous solution (iv), CUR in nanoemulsion orally, and CUR in oral suspension or methotrexate (control group). After treatment, there was a reduction in paw swelling rate, both with treatment with intravenous CUR and with methotrexate, a significant decrease in spleen and thymus weight, especially in the group treated with iv CUR, and a reduction in NF-κB activation and expression of TNF-α and IL- 1β similarly in both groups. Regarding treatment with oral CUR, the results were similar to those obtained with iv injection of CUR; however, the best results were seen in the group treated with CUR in nanoemulsion compared to suspension, suggesting the feasibility of treatment with iv CUR or oral in nanoemulsion in RA.

Arora et al. [40] evaluated the role of CUR in solid lipid nanoparticles (C-SLN) in Wistar rats RA. The animals were divided into a control group, treated with oral naproxen, oral free CUR, and oral C-SLN. After treatment, there was an increase in pain threshold, improvement in joint mobility and stiffness, decrease in paw swelling, decrease in leukocytosis, OS, and TNF-α and anti-CCP levels in the groups treated with C-SLN and naproxen similarly, while the group treated with free CUR showed no improvement in many of the aspects analyzed.

Yan et al. [41] conducted a study with Sprague-Dawley rats to evaluate the therapeutic efficacy of the nanoparticulate system composed of CUR, prednisolone, and human serum albumin (N-PRE/CUR) in models of RA. Rats with arthritis were treated with iv free prednisolone (PRE), free CUR, CUR nanoparticulate system, prednisolone and N-PRE/CUR, nanoparticle prednisolone (N-PRE), and the group treated with nanoparticle CUR (N-CUR). After the intervention, paw swelling scores were much lower in the N-PRE/CUR-treated group, and there was less pannus formation and bone destruction in this group compared to the other groups. TNF-α, IL-1β, and IL-6 were significantly reduced in N-PRE/CUR treatment compared to all other groups. At the same time, IL-10 levels were significantly increased in this group, evidencing the highest anti-inflammatory activity of N-PRE/CUR compared to free PRE, free CUR, N-PRE, or N-CUR.

In a randomized, double-blind, controlled clinical study, Javadi et al. [42] evaluated the effects of CUR on nanomicelles in patients with AR. The patients were selected after rheumatological evaluation and data such as the Disease Activity Score of 28 joints (DAS-28), tender joint count (TJC), and swollen joint count (SJC), in addition to the analysis of erythrocyte sedimentation rate (ESR). Patients were randomized into two groups, which received CUR in nanomicelles or placebo along with usual RA care. After the 12-week treatment, the DAS-28, TJC, SJC, and ESR scores were significantly reduced in both groups compared to the initial values. However, there was no statistically significant difference between the groups, although the therapeutic effect of CUR was more significant in the group treated with nanomicelles.

Studies show that CUR in nanoparticulate systems, nanoemulsions, and C-SLN can be effective therapeutic adjuvants against RA during clinical practice. CUR in different based nanomedicines could ameliorate this inflammatory and autoimmune condition via inhibiting the production and secretion of pro-inflammatory cytokines, decreasing the activation and signaling of the NF-κB, diminishing joint leukocytes migration and reducing pannus formation and bone destruction as major actions.

### 3.3. CUR-Based Nanomedicines in Osteoarthritis

Osteoarthritis (OA) is characterized by the degeneration of joint cartilage [111,112]. Inflammation is of paramount importance in the development of OA due to the production of pro-inflammatory mediators and metabolites responsible for damage to cartilage tissue. Senescence, as well as mitochondrial damage, increases ROS rates and senescence-associated secretory phenotype secretion, which act as pro-inflammatory mediators. Furthermore, the mitochondrial metabolite succinate induces the accumulation of macrophages and their differentiation into pro-inflammatory ones. Mitochondrial damage also seems to be associated with the perpetuation of the inflammatory process due to the induction of the synthesis of cytokines such as IL-1β and IL-6. However, prostaglandin E2 is primarily responsible for joint pain. Damage-associated molecular patterns (DAMPs) are released by injured cells, and these interact with receptors, perpetuating the inflammatory process. The adenosine triphosphate (ATP) released in the extracellular environment is detected by macrophages and induces the synthesis of more pro-inflammatory mediators, such as IL-1β and IL-18 [112].

Zhang et al. [43] investigated the effects of CUR in delaying the progression of OA and pain in rats as models of destabilization of the medial meniscus. Models exposed to destabilization of the medial meniscus surgery received oral or topical doses of CUR nanoparticles for eight weeks. The study pointed out as main results that the use of nanoparticles encapsulating CUR proved to be effective in reducing the expression of pro-inflammatory mediators, such as IL-1β and TNF-α, in addition to inducing the upregulation (increase in the expression of receptors) of chondroprotective transcriptional regulator CITED2 genes.

Yeh et al. [44] investigated the potential of CUR and BMC for the treatment of OA. In an in vitro study, soybean phosphatidylcholines were considered to form liposomes which were used to increase the uptake of compounds by cells. After loading the liposomes with Cur and BDMC, the particles were stable, and the encapsulation was responsible for reducing the cytotoxic effects of the substances, in addition to increasing their uptake by osteoblasts. Nevertheless, the compounds efficiently reduced the differentiation of macrophages in multinucleated tartrate-resistant acid phosphatase (TRAP) -positive cells and removed the mineral deposition in the cartilaginous tissue. Furthermore, Cur and BDMC in liposomes effectively reduced the synthesis of COX-2 and matrix MMP-3, induced by IL-1β, consequently reducing the production of pro-inflammatory cytokines.

Niazvand et al. [45] carried out an in vivo study with rats in order to evaluate the effects of CUR-loaded poly lactic-co-glycolic acid nanoparticles (nanoCUR) against OA induced by mono-iodoacetate (MIA) in the knee of the animals. The study showed the efficiency of Cur in reversing the induced cellular hypodensity. However, this effect was more evident with the use of nano CUR. Histologically, it is common for cartilage affected by OA to stain less in histological preparations due to matrix degradation. However, both Cur and nanoCUR were able to restore pigmentation, due to the induction of glycosaminoglycan synthesis, in such a way that the effect caused by nanoCUR was superior. In short, CUR effects were enhanced when it was encapsulated in nanoparticles.

Li, Stöckl, et al. [46] analyzed the effects of extracellular vesicles (EVs) in maintaining the bioavailability and stability of CUR against OA. The authors evaluated the effects of CUR EVs in cellular models of IL-1β stimulated human osteoarthritic chondrocytes and observed that CUR EVs potentiated viability and reduced IL-1β-induced apoptosis of cells while reducing the inhibition of cell migration caused by IL-1β. In short, Cur-EVs have the ability to attenuate the catabolic processes stimulated by IL-1β while promoting the modulation of critical anabolic effects in the treatment of OA.

In an in vitro study, Kang et al. [47] used Poly(β -amino ester) (PAE), an amphiphilic polymer, to carry and increase the bioavailability of CUR to measure its effects on OA induced by MIA in the knee of rats. They observed that RAW264.7 macrophages almost immediately internalized the micelles, therefore improving compound uptake, whereas no relevant cytotoxic effect was observed in either RAW264.7 macrophages or chondrocytes. After stimulating the generation of ROS induced by hydrogen peroxide (H_2_O_2_) or lipopolysaccharides (LPS), the use of CUR micelles was more effective compared to free CUR in reducing the toxicity of both H_2_O_2_ and LPS. There was also suppression of TNF-α expression and upregulated expression of IL-1β to levels beyond those achieved by CUR in its free form.

Crivelli et al. [48] used silk fibroin nanoparticles (SFNs) obtained from Bombyx mori cocoons to encapsulate celecoxib (CXB) and CUR in OA models. The study shows that CUR encapsulation is an interesting alternative in the treatment of OA due to its lower cytotoxicity when compared to CXB.

The role of CUR-based nanomedicines also gains space during musculoskeletal system inflammatory and autoimmune conditions, as in the case of RA. Due to the above information, we can say that CUR in the forms of nanoparticles, liposomes, CUR-loaded poly lactic-co-glycolic acid nanoparticles, EVs, PAE, and SFNs were effective in decreasing OA manifestations throughout decreases in pro-inflammatory cytokines production and secretion, reductions in oxidative parameters, induction of glycosaminoglycan synthesis, reductions in MMPs synthesis and secretion and upregulation of chondroprotective transcriptional genes as major actions.

### 3.4. CUR-Based Nanomedicines and Neurodegenerative Diseases

Neurodegenerative diseases (ND) are brain disorders characterized mainly by progressive loss of selectivity in vulnerable populations of neurons, which contrasts with metabolic or toxic brain disorders due to the select static neuronal loss that occurs in these. Theoretically, ND can be classified according to primary clinical signals such as dementia, motor neuron disease or parkinsonism, anatomic distribution of neurodegeneration such as frontotemporal degenerations, extrapyramidal disorders or spinocerebellar degenerations or, principally, ND can be classified as their molecular abnormality. Nowadays, the most popular ND are Alzheimer’s and Parkinson’s diseases [113,114,115].

#### 3.4.1. Alzheimer’s Disease

Alzheimer’s disease (AD) is characterized by neuritic plaques and fibrillar tangles resulting from the accumulation of amyloid beta peptide (Aβ) and massive neuronal losses. It is considered a disease of multifactorial origin due to the action of several risk factors such as advanced age, genetic factors, head trauma, vascular diseases, infections, and other factors numerous environmental factors [116,117,118]. Pathologically, the production of soluble Aβ oligomers and the activation of inflammation are the two most essential steps in AD occurrence. While the oligomers are responsible for the neuronal dysfunction proper of the AD clinical features due to misfolding protein disorders, the evidenced neurodegeneration develops neuronal inflammation, and this process must be assessed in favor of a more direct role of glial cells activation during the synaptic functions alterations [119,120].

Tayor et al. [49] studied the effects of different nanoliposomes associated with CUR and lipid ligands on the aggregation of amyloid-β 1-42 (Aβ 1-42) peptide in vitro. The nanoliposome formulations involved nanosized liposomes composed of CUR, CUR derivative, or lipid ligands such as phosphatidic acid (PA), cardiolipin (CL), or GM1 ganglioside (GM1). Nanoliposomes containing PA, CL, and GM1 showed little or no inhibitory effect on amyloid fibril formation. CUR liposomes were the most effective, showing evident concentration-dependent inhibition of Aβ aggregation.

Mathew et al. [50] investigated the role of CUR-loaded polylactic-coglycolic acid copolymer (PLGA) nanoparticles on Aβ aggregation in vitro. These nanoparticles were conjugated to Tet-1 peptide due to its affinity for neurons, facilitating the targeting of nanoparticles to the central nervous system. After co-incubation, the breakdown of amyloid aggregates was observed and breakdown into considerably smaller plaques after 48 h of coincubation. Nanoparticles conjugated to Tet-1 peptide showed similar anti-amyloid activity, although they were slower than nanoparticles without Tet-1 in formulating the same effect. Tet-1-conjugated PLGA-CUR nanoparticles also demonstrated 60% free radical scavenging activity, showing that PLGA and Tet-1 do not nullify the antioxidant property of CUR. Furthermore, the study showed that CUR-loaded PLGA nanoparticles conjugated to Tet-1 did not demonstrate in vitro cytotoxicity.

Tiwari et al. [51] analyzed the effects of PLGA nanoparticles encapsulated in CUR (Cur-PLGA-NPs) on the induction of neurogenesis and neuronal differentiation in vitro and in vivo and observed potent proliferation of endogenous neural stem cells (NSC) and neuronal differentiation in the hippocampus and subventricular zone compared to bulk CUR, increasing β-catenin nuclear translocation and increasing GSK-3β phosphorylation. These actions elevated the expression of pro-neurogenic genes. Furthermore, treatment with Cur-PLGA-NP increased proliferation even at very low doses and was not cytotoxic at high doses compared to free CUR.

Kuo & Lin et al. [52] evaluated liposomes conjugated with wheat germ agglutinin (WGA) and CL on the transport of nerve growth factor (NGF) and CUR across the blood-brain barrier and on the viability of SK-N-MC cells against apoptosis induced by Aβ 1-42 fibrils in vitro. Analyzes showed that increasing the molar percentage of CL in liposomes improved the trapping efficiency of NGF and CUR and accelerated the release of NGF from liposomes with WGA-CL-NGF-CUR. In contrast, the release of CUR from these liposomes was delayed. Furthermore, treatment with WGA-CL-NGF-CUR liposomes slightly increased the viability of SK-N-MC cells with Aβ 1-42, showing that WGA-CL-NGF-CUR liposomes can be potential colloidal delivery transporters in targeting the blood-brain barrier for AD treatment.

Fan et al. [53] analyzed the effects of CUR-loaded PLGA- polyethylene glycol (PEG) nanoparticles conjugated with B6 peptide in vitro and in vivo. In vitro results showed that all particles studied did not affect cell viability and had relatively low toxicity profiles. In vivo analyses showed that treatment with PLGA-PEG-B6/CUR improved mice’s spatial learning and memory capacity, as they spent less time finding the platform. Furthermore, treatment with PLGA-PEG-B6/CUR reduced Aβ production in the hippocampus and decreased tau phosphorylation in mice.

SoukhakLari et al. [54] experimented with the effect of bovine serum albumin (BSA) -based CUR nanoparticles on memory and concentration of MMP-2, MMP-9, and MAPKs in the hippocampus in NMRI mice. The treatment significantly optimized the performance of the mice in the passive avoidance memory test, which did not occur with the treatment with the same doses of natural CUR, showing the effectiveness of CUR nanoparticles.

Huo et al. [55] evaluated the role of selenium nanoparticles encapsulated PLGA nanospheres with CUR (Se/Cur-PLGA) on Aβ aggregation in vivo and showed that in the treated groups, the selenium nanoparticles on the surface of the nanospheres helped in the penetration of CUR through blood–brain barrier (BBB) and, consequently, in the realization of its effects. Se/Cur-PLGA nanospheres were located mainly on amyloid β plaques and completely crossed the BBB, showing that these nanospheres bound exactly to amyloid β plaques.

Zhang et al. [56] analyzed the effects of intranasally administered CUR-encapsulated chitosan-coated PLGA acid nanoparticles and CUR/hydroxypropyl-β-cyclodextrin inclusion complexes on CUR transport in AD. In vitro analysis involved human neuroblastoma cells (SH-SY5Y) and mice microglia cells (BV-2) used to evaluate cytotoxicity, cell uptake, and anti-inflammatory and antioxidant activities, while in vivo studies involved C57BL/6 mice who received CUR solution, CUR-CSPLGA-NPs or CUR/HP-β-CD inclusion complexes intranasally. The in vitro study showed that both CUR-CS-PLGA-NPs and CUR/HP-β-CD inclusion complexes showed high stability, did not alter CUR release under different conditions, and did not impair the antioxidant and anti-inflammatory activity of CUR as well as could facilitate the internalization of CUR in SH-SY5Y cells and BV-2 cells and demonstrated low cytotoxicity. The in vivo study indicated that the CUR/HP-β-CD inclusion complexes demonstrated the highest bioavailability and highest peak CUR concentration (Cmax) among the three groups, which may produce therapeutic improvement concerning the CUR solution and CUR-CS -PLGA-NPs in the long-term treatment of AD.

Lin et al. [57] evaluated the combination of PLGA-PEG-PLGA thermo-sensitive hydrogel with CUR (PGC) in preventing and decreasing the progression of AD. In vitro studies demonstrated that PGC did not exert cytotoxicity but had excellent anti-inflammatory and antioxidant properties and microglial modulation. In vivo studies demonstrated better performance of animals with AD treated with PGC injection. In addition, PGC reduced the aggregation and deposition of β-amyloid proteins in the neurons of treated mice and significantly increased hippocampal activity.

Campisi et al. [58] studied the effects of systemic administration of C-SLN on the delivery of CUR to neuronal cells and the levels of expression of the tissue transglutaminase isoform (TG2) in an experimental model of AD. After the interventions, the animals underwent a behavioral descent test to assess memory and learning. The results showed that treatment with SLNs loaded with CUR improved cognitive and memory performance, especially in Tg mice compared to WT mice. Furthermore, the different TG2 isoforms were modulated differently with systemic administration of SLNs-CUR in Tg and WT mice. TG2-L expression increased with SLNs-CUR in Tg and WT mice, whereas TG2-S expression decreased with SLNs-CUR in Tg and WT.

The study performed by Ruan et al. [59] investigated the effects of a highly-sensitive CUR-conjugated nanotheranostic platform on the reversal of cognitive deficits in AD and on the detection of Aβ plaques by magnetic resonance imaging in vivo. After treatment, all rats were evaluated for spatial reference memory by the Morris water maze (MWM) test. The results showed that this multifunctional nanomaterial efficiently reduced the Aβ plaque burden, indicating that this nanomaterial has great potential to be applied to the early diagnosis and treatment of AD.

Patil et al. [60] evaluated the applicability of a nanoimaging agent (NIA) based on poly[b-L-malic acid] (PMLA) containing gadolinium–DOTA (Gd–DOTA) and derived CUR in detecting images of Aβ plaques in human samples. Magnetic resonance imaging in the presence of NIA showed a high-contrast enhancement. At the same time, no contrast was obtained with plates incubated with free CUR and free Gd-DOTA, evidencing that NIA could bind to Aβ plates by CUR, which may be a promising method for the safe and non-invasive diagnosis of AD.

Giacomeli et al. [61] compared the effects of CUR lipid-core nanocapsules (LNC) and free CUR (FC) in a model of induced AD in aged Swiss Albino mice. After 14 days, treatment with FC and LNC reduced the cognitive deficit induced by Aβ 1-42 in the MWM test. Furthermore, the administration of Aβ 1-42 increased the mRNA expression of TNF-α, IL-1β and IL-6, IFN-γ and NF-κB in the hippocampus and prefrontal cortex of control mice, while treatment with FC and LNC decreased expression of TNF-α, IL-1β, IL-6, IFN-γ, and NF-κB mRNA.

The above information shows that CUR-based nanomedicines such as liposomes, nanoparticles, nanocapsules, and nanospheres can promote anti-dementia effects and protect against AD. The results of the included studies evidenced that CUR in the form of nanomedicines decreased Aβ aggregation, amyloid fibril formation and TG2 expression, upregulated Aβ aggregates breakdown, neurogenesis, neuronal differentiation, the proliferation of endogenous NSC, β-catenin nuclear translocation, GSK-3β phosphorylation, expression of pro-neurogenic genes, neuronal cells viability, spatial learning, memory capacity, and microglial modulation, as well as reduced Tau protein phosphorylation, mRNA expression of TNF-α, IL-1β and IL-6, IFN-γ and NF-κB and brain oxidative stress.

#### 3.4.2. Parkinson’s Disease

In turn, Parkinson’s disease (PD) causes movement disorders. The pathological hallmark of PD consists of neural inclusions in the form of Lewy bodies and Lewy neurites, with selective degeneration of dopamine neurons in the substantia nigra, with decreased levels of dopamine in the striatum and other areas of the brain, which leads to impaired motor control [121,122,123]. In PD, inflammation is also part of its pathogenesis. During PD, principally, the neuronal death of dopaminergic neurons from the substantia nigra *pars compacta*, and the consequent microglial cell activation leads to the expression of several pro-inflammatory cytokines that implicates degeneration on even more dopaminergic neurons. Different from other ND, the gut–brain axis and the possible contribution of dysbiotic-bowel peripheral inflammation could also contribute to the brain’s neuroinflammation and, therefore, to the death of neurons [124,125].

Bollimpelli et al. [62] studied the neuroprotective effects of CUR-loaded lactoferrin nanoparticles on rotenone-induced PD in dopaminergic cell line SK-NSH pretreated with CUR in solution, nanoCUR equivalent or lactoferrin nanoparticles (deprived of CUR), in addition to being subsequently treated with rotenone for induction of neurotoxicity. The assays showed that pretreatment with CUR or nanoCUR solution rescued cells from rotenone-induced neurotoxicity but more substantially in pretreatment with nanoCUR, while lactoferrin nanoparticles devoid of CUR showed no significant effect in attenuating neurotoxicity. In addition, ROS levels were reduced with pretreatment with CUR and nanoCUR solution. The expression of Tyrosine Hydroxylase (TH), an enzymatic marker of dopaminergic cell injury, was efficiently retained with nanoCUR pretreatment. In contrast, the expression of α-synuclein, a critical component in Lewy body formation, was also deleted.

Zhang et al. [63] evaluated the role of CUR-loaded modified polysorbate 80 cerassome (CPC) nanoparticles (NPs) in the localized delivery of CUR to targeted brain nuclei through an adequate opening of the BBB by ultrasound-targeted microbubble destruction (UTMD). The results showed that treatment with CPC NPs combined with UTMD in MPTP-induced PD mice notably improved behavioral disturbance, dopamine depletion, and TH expression, as well as reduced α-synuclein (AS) expression, showing that the delivery of CUR with this treatment was efficient, as well as its therapeutic effects against PD.

Sookhaklari et al. [64] evaluated the effect of BSA-based nanoCUR against 6-OHDA-induced cell death in vitro. SH-SY5Y cells were treated with OHDA and subsequently with different doses of nanoCUR and CUR free. Treatment with nanoCURA at doses of 400 and 500 nM prevented cell death. Furthermore, this CUR nanoformulation restored the 6-OHDA-induced p-Akt/t-Akt decrease. This neuroprotective effect of CUR was four times higher with CUR in nanoformulation compared to CUR free.

Liu et al. [65] experimented with a peptide-modified exosome chemical complex (EXO) CURa/phenylboronic acid-poly(2-(dimethylamino)ethyl acrylate) nanoparticle/small interfering RNA targeting SNCA (REXO-C/ANP/S) as a nano scavenger to remove aggregates of α-synuclein and reduce its cytotoxicity in PD neurons. C57BL/6 mice received MPTP for PD induction, and then REXO-C/ANP/S treatment and other control NPs were administered. After treatment, mice from the NP groups showed a trend toward improvement in exercise, especially the REXO-C/ANP/S group. The neuronal repair was also superior in PD mice injected with REXO-C/ANP/S compared to the other groups. Furthermore, treatment with REXO-C/ANP/S was more effective in clearing α-synuclein, reduced IL-2 and IL-17 expression, and increased IL-10 expression.

Yan et al. [66] studied the role of PLGA-lipid nanobubbles (NBs) in the delivery of CUR and BBB opening induced by low-intensity focused ultrasound (LIFU) in C57BL/6 mice. After treatment, the rats were subjected to behavioral tests to assess the remission of PD symptoms. Performance on the behavioral test among mice-induced PD was superior in mice treated with Cur-NBs combined with LIFU. Furthermore, Cur-NBs combined with LIFU improved the local delivery of CUa to deep brain nuclei, significantly potentiating the effectiveness of CUR compared to groups that were treated with either Cur-NBs alone or LIFU alone.

Furthermore Guzman et al. [67] analyzed the effect of CUR-loaded human serum albumin nanoparticles (CUHNP) on the prevention of PD-like symptoms in *Caenorhabditis elegans*. Treatment with CUHNP effectively delayed the deterioration of nematode movement. Ultimately, CUHNP could enhance the activity of dopamine transporters at the end of presynaptic neurons, resulting in enhanced dopamine transport to synaptic neurons.

Due to the above information, CUR-based nanomedicines are effectively more efficient as an adjuvant treatment against PD than free CUR. CUR in the form of NPs, exosomes, and NBs exerted reduction in α-synuclein expression, brain OS, TH expression, Lewy body formation, behavioral disturbances, dopamine depletion, neuronal cells death and IL-2 and IL-17 levels, as well as increases in neuronal repair, IL-10 levels and dopamine transport to synaptic neurons. These actions can contribute highly to anti-PD treatment clinically.

#### 3.4.3. Multiple Sclerosis

Multiple sclerosis (MS) is a complex neurodegenerative disease characterized to be an inflammatory process with the production and release of pro-inflammatory cytokines that also lead to oxidative burden. This pathophysiological inflammation and OS result in demyelination, reduced remyelination, and decreased axonal survival, together with massive activation of microglial cells. As a substantial share of patients suffering from MS present deterioration of neurological functions slowly and considering the silent progression of the disease, the use of anti-inflammatory and antioxidants compounds is gaining interest towards its easy manipulation, many benefits, and pleiotropic activity with few cytotoxicity as adjuvants in MS treatment [126,127].

Motavaf et al. [68] investigated the effects of dendrosomal CUR nanoparticles (DNCur) on oligodendrogenesis and remyelination both in vitro and in vivo using models of MS demyelination. The results indicated that DNCur effectively enhanced oligodendrogenesis from NSC and oligodendrocyte progenitor cells in a dose-dependent manner in vitro. The CUR-based nanomedicine also promoted remyelination via promoting oligodendrogenesis in vitro. On the other hand, in vivo, DNCur had a significant impact in enhancing the remyelination capacity of transplanted NSC through the promotion of not only their survival but also oligodendrogenesis enhancement.

Motavaf et al. [69] also conducted an in vivo study to evaluate the protective effects of DNCur against cuprizone-induced (CPZ) demyelination in the mouse corpus callosum. The results showed that the use of DNCur protected the oligodendrocyte lineage cells against CPZ-derived demyelination, as well as suppressed the accumulation of astrocytes and microglia cells in the *corpus callosum* of the CPZ-fed mice. In addition, the DNCur treatment also increased the index of luxol fast bluefast blue and myelin-specific proteins as an indicator of myelin content, as these are myelin-specific proteins. The authors, therefore, suggested an efficient pleiotropic therapeutic strategy for DNCur in the myelinating protection of cells via possibly suppressing astrocytes and microglia.

Lu et al. [70] conducted an in vivo study with a mice model of experimental autoimmune encephalomyelitis and found that targeted immunomodulation of inflammatory monocytes across the blood-brain barrier by CUR-NPs was an effective strategy to not only augment CUR bioavailability but also delay the progression of that MS model. The authors used a high-density lipoprotein-mimicking peptide-phospholipid scaffold (HPPS) as a way to ameliorate CUR bioavailability and to create CUR-loaded HPPS (CUR-HPPS) NPs that were taken specifically and efficiently by inflammatory monocytes through their scavenger receptor B type 1 (SR-B1). After this taking, the monocytes were infiltrated across the blood-brain barrier of the encephalomyelitis mice. The liberation of CUR resulted in decreased microglia proliferation, restricted immune cell infiltration in the neuronal areas, and reduced morbidity of the experimental model from 100% to 30%. Molecularly, CUR attenuated NF-kB activation and inhibited the expression of adhesion and migration-related molecules in the mice’s brain.

Dolati et al. [71] conducted a clinical study and found that the use of nanoCUR improved regulatory T-cell (Treg) frequency and function in patients with MS. In all, 50 patients were enrolled in this trial, and 25 of them were treated at least for six months with preparations of nanoCUR capsules while the others received placebo. The results revealed a decrease in the proportion of peripheral Treg cell frequency and the levels of TGF-β, IL-10, and forkhead box protein 3 (FoxP3) expression. It has been discovered that the disturbance in the development and function of Treg subpopulations could be associated with disabilities in most patients with MS. The use of nanoCUR, therefore, can be a pathway in the restore of the frequency and function of Treg cells in these patients.

Dolati et al. [72] also evaluated in what appears to be the same population as of the previous study the effects of nanoCUR as a potent anti-inflammatory treatment or adjuvant against MS. The authors found that nanoCUR was able to decrease the expression levels of the inflammatory miR-145, miR-132, and miR-16. In addition, nanoCUR decreased signal transducer and activator of transcription (STAT) 1, NF-kB, and AP-1 activation and signaling in MS patients, as well as increased STAT5 mRNA expression levels. The use of nanoCUR also reduced the levels of IL-1β, IL-6, chemokine (C-C motif) ligand (CCL) 2, CCL5, IFN-γ, and TNF-α mRNA expression levels.

Due to the above results, it is possible to say that CUR-based nanomedicines are effective agents against demyelination during MS treatment. CUR in the forms of dendrosomal CUR NPs, CUR-HPPS, and simple nanoCUR were able to increase oligodendrogenesis, remyelination, neuronal myelin content and STAT5 mRNA expression levels in patients and models with MS. Additionally, the nano formulations with CUR decreased astrocytes and microglia cells accumulation and actions, microglial proliferation, disease’s morbidity, NF-kB activation and signaling, adhesion and migration-related proteins, peripheral Treg cell frequency and function, TGF-β, IL-10 and FoxP3 expression levels, inflammatory miR-145, miR-132, and miR-16 expression levels and STAT1 activation and signaling, as well as the expression levels of IL-1β, IL-6, CCL2, CCL5, IFN-γ and TNF-α mRNA.

#### 3.4.4. Huntington’s Disease

Huntington’s disease (HD) is a neurodegenerative condition caused mainly by an abnormal expansion of polyglutamine replicated in the first exon of the huntingtin gene. The disease is characterized by neurodegeneration, particularly in the brain’s striatum and cortex. The mutation in the huntingtin causes abnormalities in the functioning of the codified protein, which leads to deleterious effects, ultimately to the demise of specific neurons and other neuronal cells. The first HD symptoms and signals appear in mid-life and include cognitive deficits and motor disturbances in a progressive timeline. Although this disease is inherited, treatments have been proposed over the years, and many of them significantly improve HD patients’ quality of life [128,129]. Despite synthetic approaches, CUR-based therapies have been proposed against HD due to the anti-aging and anti-neurodegeneration effects of this polyphenol [130,131,132]. However, CUR-based nanotherapies usually obtain better results against HD as well.

Traditionally, researchers focused on brain changes as the cause of progressive motor and cognitive dysfunction during HD. However, the discovery of huntingtin protein and its mutated form being expressed in different organs and tissues than the brain corroborated the hypothesis that other mechanisms could be involved in HD disease, like an inflammatory response. New research evidenced that inflammatory states can be evaluated since the onset of classical HD signals and symptoms as inflammation can adjuvantly cause systemic suppression of energy metabolism, failure of transcription, and mitochondrial dysfunction, as well as contributes to abnormalities of neurons’ cytoskeleton, microglial disruption, and impairments to the axonal neuron transport [133,134,135,136].

Sandhir et al. [73] discussed in an in vivo study with a mouse model of HD the effects of CURNPs in neurochemical and neurobehavioral deficits encountered during the disease. Truly, the authors wanted to explore the effects of the CUR-based nanotherapy against the typical HD mitochondrial dysfunction. CUR was encapsulated in C-SLNs and was used to ameliorate 3-nitropropionic acid (3-NP)-induced HD in rats. The results showed that CUR significantly penetrated the animals’ brain and decreased the striatum’s Complex II activity. However, the treated animals also presented elevated mitochondrial complexes activity, as well as increased cytochrome levels. Molecularly, CUR diminished the oxidative stress imposed on the rats’ brains due to a significant increase in GSH and SOD levels and decreased mitochondrial swelling, lipid peroxidation, protein carbonyl formation, and ROS production. At least, the mice presented significant improvements in neuromotor coordination. These effects were attributed to the activation of the Nrf2 by CUR.

### 3.5. CUR-Based Nanomedicines in Epilepsy

Epilepsy is a brain condition characterized by the recurrence of unprovoked seizures. In general, epilepsy prognosis refers to the probability of attenuating the recurrence of the seizures, increasing seizure freedom on treatment. Seizures occur mainly when an abnormal synchronous neuronal firing in a section of the brain happens, as well as throughout the entire brain or when the brain’s networks begin irregularly formed or are perturbated by any structural, infectious, or metabolic disturbance. Practically, the differences between etiologies among the sexes and age groups result in diagnostic difficulties for all clinicians [137,138]. The benefits of free CUR-based therapies against epilepsy have been studied in the past [139,140]. However, the use of CUR-based nanotherapies revolutionized the area due to, again, the increase in CUR’s bioavailability to the central nervous system.

The basic pathophysiology of epilepsy is not completely understood. However, large evidence suggests that this condition is not only a neurological disease but also a systemic disorder and that the pathophysiological process involves inflammation [141]. Chronic brain inflammation comprising the activation of microglia astrocytes, peripheral immunological cells, and endothelial cells of the blood-brain barrier promotes the production and release of pro-inflammatory mediators that are evidenced in many types of seizures. In addition, the high incidence of seizures among patients with autoimmune diseases and encephalitis also corroborates the theory that inflammation is typically associated with seizure occurrence [142]. The differentiation between immunological dysfunction-derived seizures and intrinsic brain inflammation seizures is still not commonly done, and the published data are from autopsies. Chronic brain inflammation leading to seizures may be derived from neuronal cell loss in many neurological diseases. Neuropathies leading to abnormal cerebral excitability, and enhanced-induced neuropathologies, are also in the seizure-basic pathophysiology [143,144,145].

Huang et al. [93] studied CUR C-SLN to evaluate the neuroprotective effects of CUR against epilepsy in a mice model of this condition. The results showed that CUR protected the animals against epilepsy through the activation of the B-cell lymphoma 2 (Bcl-2) family progenitors and the P38 MAPK pathways. In addition, the results also demonstrated that the CUR lipid NPs were more efficiently transported through the blood-brain barrier than free CUR, as well as ameliorated more significantly the behavioral performance of the epileptic mice by reducing neuronal apoptosis and protecting the brain against OS. In vitro, the authors also confirmed that CUR lipid nanoparticles functioned better than free CUR against neuronal apoptosis, all well as against OS.

Mansoor et al. [94], using an experimental model of chronic epilepsy, evaluated the neuroprotective roles of CUR-loaded NPs against that neuronal condition. The authors experimented with the pentylenetetrazol (PTZ)-induced kindling animal model and affirmed that CUR significantly protected against epilepsy via upregulation of klotho and erythropoietin (EPO), as well as via the reduction of neuronal cell death. Furthermore, CUR effectively down-regulated the levels of TNF-α mRNA among the CUR-receiving animals, contributing to the neuroprotective effects of this phenolic compound against chronic epilepsy.

Hashemian et al. [95] studied the effects of CUR-loaded chitosan-alginate sodium tripolyphosphate (STPP) NPs against memory deficits and reduced glial activation in the pentylenetetrazol-induced kindling model of epilepsy. Behavioral results showed that the CUR NPs were significantly associated with anticonvulsant activity and prevented cognitive impairment in fully kindled animals. CUR NPs also significantly reduced neuronal cell death and glial activation than free CUR among diseased mice.

CUR-based nanomedicines can be an effective adjuvant treatment against this brain disorder. The results showed that CUR solid lipid NPs, CUR-loaded NPs, and CUR-loaded chitosan-alginate STPP NPs could effectively increase brain Bcl-2 family progenitors’ activation, P38 MAPK pathways activation, behavioral performance, klotho levels and EPO levels, as well as reduce neuronal apoptosis, neuronal OS, TNF-α mRNA levels, microglia inflammatory activation, and memory deficits among models of epilepsy.

### 3.6. CUR-Based Nanomedicines in Inflammatory Bowel Diseases (IBD)

**Figure 2:**
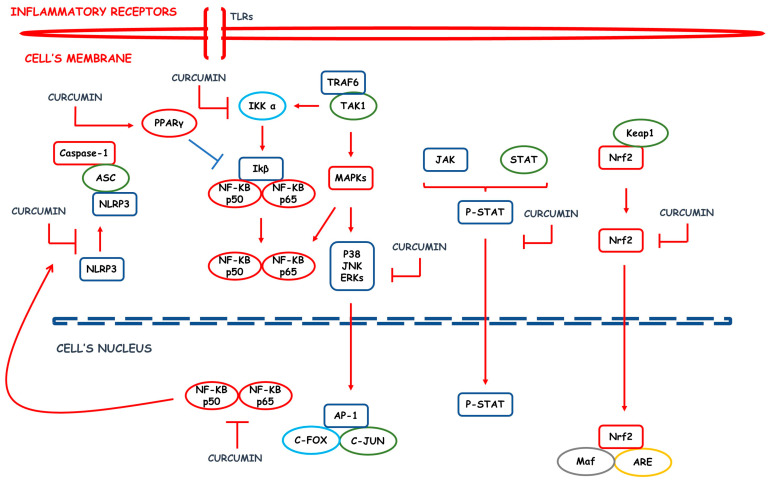
Most important pro-inflammatory vias involved in the occurrence of inflammatory bowel diseases that are inhibited by CUR. This bioactive compound diminishes inflammation and promotes antioxidant effects during in vivo, in vitro, and human studies regarding its use against IBD. The inhibition of NF-kB activation by CUR is highlighted. Ap1, apetala type 1; ARE, antioxidant redox element; ASC, apoptosis-associated speck-like protein; C-FOX, forkhead box protein C; C-JUN, transcription factor Jun C; ERK, extracellular signal-regulated kinases; IkB, nuclear factor of kappa light polypeptide gene enhancer in B-cells inhibitor; IKK a, IκB kinases α; JAK, Janus kinase; JNK, N-terminal kinase; Keap1, kelch-like ECH-associated protein 1; Maf, MAF BZIP Transcription Factor; MAPK, mitogen-activated protein kinase; NF-kB, nuclear factor kappa b; NLRP2, NLR family pyrin domain containing 3; Nrf2, nuclear factor erythroid 2–related factor 2; P38, p38 signaling transduction pathway; P50, NF-kB P50 heterodimer; P65, NF-kB P65 heterodimer; PPARγ, peroxisome proliferator-activated receptor γ; P-STAT, phosphorylated STAT; STAT, signal transducer and activator of transcription; TAK1, transforming growth factor beta-activated kinase 1; TLRs, tool-like receptors; TRAF6, TNF receptor-associated factor 6.

Inflammation in the gastrointestinal tract and chronic or recurrent immune activation characterize gastrointestinal inflammatory bowel diseases (IBD). The two main types of IBD are Crohn’s disease (CD) and ulcerative colitis (UC) [146]. CD consists of chronic inflammation and can affect any portion of the alimentary tract, emphasizing the distal regions of the small intestine and proximal areas of the large intestine. Furthermore, it comprises a discontinuous inflammatory condition along the longitudinal axis of the intestine that can affect all intestinal layers, from the mucosa to the serosa. In turn, UC has its manifestation restricted to the large intestine. The etiology of IBD is still unknown, but the most accepted hypothesis is that it is multifactorial, encompassing genetic, immunological, and environmental causes [3,147]. Figure 2 summarizes the most important pro-inflammatory vias involved in the occurrence of IBD that are inhibited by CUR.

Huang et al. [74] postulate that one of the most significant barriers in treating UC with NPs is their ability to penetrate the intestinal mucosal barrier. Thus, the authors encapsulated CAT and CUR in PLGA-based nanoparticles (PLGA-NPs). Raw 264.7 macrophages treated with LPS were used to measure the anti-inflammatory and antioxidant capacity of PLGA-NPs. Penetration of particles coated with PF127 was more effective than without PF127. Nevertheless, the release of CUR was potentiated by CAT. Furthermore, P-CAT/CUR-NPs inhibited the release of TNF-α by macrophages, in addition to causing an increase in IL-10 concentrations and a drastic reduction in ROS.

C. Liu et al. [75] investigated the therapeutic potential of turmeric-derived NPs, specifically in a population of NPs with a hydrodynamic size of 178 nm and a high amount of CUR, called TDNPs 2, in the treatment of UC. The in vitro culture consisted of colon-26 cells and a population of RAW 264.7 macrophages, while the in vivo models were FVB/NJ mice and NFκB-RE-Luc transgenic mice. To evaluate the effects of TDNPs 2 on macrophages, a model of inflammation induced by LPS was used, in which a large reduction in the levels of pro-inflammatory cytokines such as TNF-α, IL-1β, and IL -6 was observed. In the in vivo study, rats were given 3% (*w*/*v*) dextran sulfate sodium (DSS) to induce and stabilize colitis. Tests have shown a preferential rate of TDNPs 2 to the colon’s inflamed regions, highlighting the anti-inflammatory and antioxidant properties of these CUR-rich NPs by suppressing mediators such as TNF-α, IL-6, and IL-1β, as well as the inhibition of the NF-κB pathway. Nevertheless, the CUR-rich treatment stimulated the expression of antioxidants such as HO-1.

Li et al. [76] used an enzymatic trigger to release CUR in the form of CUR-cyclodextrin (CD-Cur) protected by a chitosan capsule and unsaturated alginate resulting in nanoparticles (CANPs). For the in vitro model, macrophages (RAW 264.7) were used, while C57BL/6 mice were used as a model of UC. The in vitro study demonstrated that the uptake of the compound encompassed by CANPs was practically integral. In reaction with the stomach acid environment, the carboxyl group of alginates and the amino group of chitosan formed a gel that protected the active principle from the aggressive stomach microenvironment of in vivo models. The release of the biocomponent was stimulated in the simulated ileum microenvironment (pH 7.4), which was further potentiated in the presence of α-amylase. The analysis of the gastrointestinal tract of the animals used in the experiment showed the affinity of CD-Cur-CANPs for the portion of the colon where they accumulated and demonstrated significant biodistribution. Furthermore, CD-Cur-CANPs were efficient in preventing weight loss in the group treated, in addition to reversing the reduction in colon length and the increase in spleen size.

C. Wang et al. [77] proposed in vitro and in vivo models to study the potential of liposomes to encapsulate and ensure that CUR reaches the site of action. In vitro studies have shown that liposomes effectively protect the CUR from the simulated stomach fluid environment. The animals treated with CUR-LPs, in turn, showed significant improvements in weight loss, intestinal bleeding, and diarrhea, when compared to those treated only with the aqueous solution of CUR. Furthermore, CUR-LPs proved effective in inhibiting the production of pro-inflammatory cytokines, such as TNF-α and IL-6. Thus, CUR-LPs can be an important therapeutic alternative for promoting drug resistance to the hostile stomach microenvironment, in addition to allowing the release under the action of intestinal lipases and facilitating the uptake of CUR in the intestinal environment.

Oshi et al. [78] evaluated the transport and release of CUR nanocrystals along the GI tract when encapsulated by multiple layers of chitosan (CH), sodium alginate (AG), and cellulose acetate phthalate (CAP), as well as their advantages for the treatment of UC. Better results were noted in complexes with ten CAP/AG/CH layers, which were more efficiently distributed in the colon than the others. The model CUR nanocrystals surrounded by CH (CH1@CUNCs) in the form CAP1AG4CH5@CUNCs proved to be more efficient in accumulating in the inflamed intestinal tissue, even reducing the levels of infiltrated neutrophils and macrophages, in addition to consequently decreasing TNF-α and IL-6 levels.

X. Zhang et al. [79] produced NPs loaded with CUR and conjugated their surfaces with chondroitin sulfate (CS) to evaluate drug delivery to macrophage colonies present in the intestinal mucosa under UC when compared to its counterpart coated with cellulose. The in vitro study used RAW 264.7 macrophages, and the in vivo study used mice; the PNs were soaked in a hydrogel based on CH and alginate to protect them from the acidic and aggressive microenvironment of the stomach. After incubation for 24 h with RAW 264.7 macrophages and CT-26 cells, the CS-NPs were not cytotoxic, in addition to presenting good biocompatibility, allowing a high rate of internalization by macrophages. In animals, the hydrogel proved to be efficient in protecting NPs during their passage through the stomach, allowing the drug to reach the intestine. Nevertheless, the uptake rate by macrophages was higher for NPs coated with CS than for those coated with carboxymethyl cellulose. Furthermore, the group treated with CS-NPs showed significant improvement in terms of weight loss when compared to the UC control group or even that treated with CUL-NPs, thus confirming the therapeutic potential of CS-NPs.

Hlaing et al. [80] proposed using PGLA loaded with CUR and coated or not with hyaluronic acid (HA). Colon evaluation of UC model animals showed that NPs coated with HA were more successful in accumulating in the inflamed tissue due to overexpressed CD44 receptors; therefore, adherence of NPs was shown to be high by the HA coating, as well as the cellular uptake and intracellular encapsulated drug release, consequently. In vitro tests showed similar release profiles for CUR-PLGA-NPs and CUR-HA-PLGA-NPs after 48 h of incubation. Both treated groups showed better evolution than the untreated ones. However, the group that used CUR-HA-PLGA-NPs showed a more satisfactory body weight recovery and a higher survival rate when compared to the others. Furthermore, the rats that received CUR-HA-PLGA-NPs had reduced levels of pro-inflammatory cytokines (TNF-α and IL-6).

Chen et al. [81] studied porous NPs of CUR-loaded PLGA with pluronic F127 (PF127). In vitro studies demonstrated that, when exposed to a pH of 7.4, all NPs showed similar release profiles of the CUR compound (fast initial release, followed by a slower and constant release phase), with porous NPs showing faster release than non-porous ones. The pattern was maintained in the simulated colonic environment (pH 6.2), in such a way that the porous PF127-NPs showed a higher rate of compound release. Furthermore, the PF127-NPs proved efficient in protecting the substance internalized in the acidic environment of the simulated gastric fluid, with a release rate of only 28%. The study using RAW 264.7 macrophages showed that the uptake of CUR by these cells was promoted by coating the vesicles with PF127, although the nonporous PF127-NPs were more efficiently captured. The anti-inflammatory properties (inhibition of TNF production -α and IL-6, and stimulation of the IL-10) were more pronounced in porous NPs, especially those coated with PF127. The in vivo study showed that adding PF127 to the surface of NPs potentiates their accumulation in inflamed intestinal tissue. Thus, NPs coated with PF127 can be effective in enhancing the beneficial effects of CUR in the treatment of UC and reducing the compound’s toxicity.

Arafat et al. [82] investigated the effects of nanoCUR (NC) on telocytes (TCs), interstitial cells that, in the colon, form the subepithelial reticular network, responsible for supporting the cells of the intestinal crypts and participating in tissue repair and regeneration processes. The analysis showed that the use of NC can be a therapeutic alternative for the treatment of UC since the compound proved to be efficient in inhibiting pro-inflammatory mediators, as well as favoring the maintenance of tissue architecture.

Conclusively, CUR-based nanomedicines are valuable as adjuvant treatment strategies for IBD due to the reductions in TNF-α, IL-1β, IL-6, and ROS levels, weight loss, intestinal bleeding, diarrhea, infiltrated neutrophils, and macrophage levels. In addition, CUR-based nanomedicines induced IL-10 and HO-1 production, as well as decreased expression of NF-kB.

### 3.7. CUR-Based Nanomedicines in Psoriasis

Psoriasis is a chronic inflammatory skin disease whose etiology is strongly associated with genetic predisposition and autoimmunity [148]. This is characterized by a presentation of erythematous, scaly, pruritic, and well-defined plaques, especially on the trunk and the extension surfaces of the limbs and scalp. Nail, joint, and systemic involvement, with an increased risk of developing metabolic syndrome, chronic kidney disease, and cardiovascular disease, are considerably related to psoriasis [149]. Dysfunctions in the innate and adaptive cutaneous immune response are the prelude to the development and maintenance of psoriatic inflammation, which results in the uncontrolled proliferation of keratinocytes, the presence of acanthosis and dermal inflammatory infiltrates typical of the disease [150]. One of the proposed immunological mechanisms involves the recognition of antimicrobial peptides (AMPs), such as LL37, which are secreted by damaged keratinocytes and are characteristically overexpressed in psoriatic skin. These stimulate TLR9 in plasmacytoid dendritic cells that release type I IFN, which promotes the maturation of myeloid dendritic cells and differentiation of T lymphocytes into Th1 and Th17 subtypes and the consequent release of cytokines IFN-γ, IL-17, IL-21, and IL-22 activators of keratinocyte proliferation in the epidermis [151,152].

Y. Zhang et al., 2019 [83] evaluated the efficacy of propylene glycol-based ethosomes (ES) modified with HA (HA-ES) and loaded with CUR in the topical treatment of imiquimod-induced psoriasis (IMQ) in C57BL/6 mice with psoriatic inflammation. After the intervention, the analysis showed that the retention of CUR in psoriatic skin with HA-ES was 2.3 and 4.0 times greater than that of ES and PGS, respectively. In addition, the HA-ES group loaded with CUR showed relief of inflammatory symptoms according to the clinical psoriasis area and severity index (PASI), lower levels of TNF-α, IL-17A, IL-17F, IL-22, and IL-1β mRNA, and lower CCR6 protein expression compared to ES and PGS groups.

Rapalli et al., 2020 [84] observed the effects of CUR-loaded nanostructured lipid carriers (NLC) on optimizing CUR delivery for the treatment of psoriasis in vitro and ex-vivo. The results showed that CUR-NLC showed prolonged in vitro release of up to 48 h, while free CUR showed 100% drug release in just 4 h. Ex-vivo skin permeation studies revealed 3.24-fold improved skin permeation and retention in the CUR-loaded NLC gel treatment compared to free CUR gel, suggesting that the NLC-based formulation has the potential to improve CUR efficacy.

Gomez et al., 2019 [85] evaluated the combination of CH/alginate NPs loaded with CUR (Cur-CS/Alg NPs) and blue light-emitting diode (LED) light irradiation in suppressing TNF-α-induced psoriatic activity in vitro. Human HaCaT keratinocytes were cultured and incubated with TNF-α. Free CUR dissolved in dimethyl sulfoxide (DMSO) and Cur-CS/Alg NPs were added to the cells at final CUR concentrations of 0.05 and 0.1 µg/mL, while DMSO and CS/Alg NPs were used as controls. After treatment, analyzes showed that CS/Alg NPs were not toxic to normal HaCaT cells, while 0.05 µg/mL and 0.1 µg/mL of free CUR and Cur-CS/Alg NPs inhibited hyperproliferation of HaCaT cells with psoriatic inflammation induced by TNF-α. However, Cur-CS/Alg NPs showed better potential than free CUR, especially when combined with blue LED light irradiation.

Algahtani et al., 2020 [86] evaluated a nanuemulgel-based delivery system of combined CUR IMQ (IMQ-CUR-NEG) in minimizing psoriasis-like cutaneous adverse reactions in vivo. BALB/c mice were divided into three different groups according to treatment: a group treated with IMQ gel, a group treated with IMQ in nanoemulgel (NEG), and a group treated with IMQ-CUR-NEG for ten consecutive days. After treatment, the combined NEG system of CUa and IMQ completely prevented the appearance of psoriasis-like lesions compared to the IMQ-NEG and IMQ gel formulations. In addition, IMQ-NEG delayed and reduced psoriasis-like skin reaction compared to conventional imiquimod gel, which was attributed to the gradual release property of the nanoencapsulated delivery system.

In a randomized, double-blind, placebo-controlled phase III clinical trial, Bilia et al., 2018 [87] evaluated the effect of CUR NPs in patients with moderate to severe psoriasis. After treatment, the results showed that the group treated with the combination of acitretin plus oral CUR NPs performed better than the group treated with acitretin alone in decreasing PASI.

According to the above information, CUR-based nanomedicines in the forms of CUR-loaded HA-ES, CUR-loaded NLC, CUR-loaded Cur-CS/Alg NPs, CUR NEG -based delivery system, and simple CUR NPs were effective as adjuvants during psoriasis treatment. The nanoformulations of CUR were majorly able to decrease psoriasis inflammatory symptoms, PASI, TNF-α, IL-17, IL-22 and IL-1β levels, C-C chemokine receptor type 6 (CCR6) expression, the proliferation of psoriatic cells and occurrence of psoriatic lesions.

### 3.8. CUR-Based Nanomedicines in Liver Fibrosis

Fibrosis is the increase in the amount of fibroblasts and disorganized collagen deposition. In situations in which parenchymal repair is impaired, there is an increase in the connective stroma that allows the organ to function, however, with a relative loss of functional capacity. Fibrosis derives from the healing of a previous injury or even from reactional processes that are not related to the repair of the extracellular matrix [11,12].

The first stage of fibrosis resides in the inflammatory answer. Upon aggression, there is the release of cytokines and growth factors that stimulate the production and deposition of collagen in the organ. The aggressions have varied etiologies and may originate from viral agents, parasites, auto-immune response, or even external factors to the organ, as in the case of angiotensin II in myocardial fibrosis in patients with systemic arterial hypertension. From the presence of inflammatory mediators, fibroblasts are taxied to the injured site, and as a result of other substances, they proliferate and initiate the deposition of a collagenous matrix in the tissue. The mediators involved in the proliferation, differentiation, and activation of fibrosis-activated fibroblasts are diverse and highly dependent on the tissue and organ in which the repair is being performed. However, cytokines such as TNF-α, IL-6, PDGF, and IL-4 and chemokines such as CCL2 and CCL4 are important and common mediators in the proliferation and differentiation processes of this cell type in different locations. Among the most critical growth factors, TGF-β and IL-13 are essential in the fibrotic process. The immune system also plays a unique role in repair events, and M2 macrophages are the ones that most participate in the fibrotic process due to their role in the production of PDGF and TGF-β [11,12,153].

In the case of localized liver tissue necrosis, hepatocytes can regenerate. However, in cases of extensive damage, the matrix is compromised, which is a fundamental structure for the differentiation and organization of hepatocytes. In this scenario, there is the formation of a fibrous scar. However, in situations where the damage is even more pronounced, the involvement of the matrix results in the collapse of the lobular structure of the liver, which, once extensively scarred, gives rise to the condition of cirrhosis of the liver [11,12]. Alcohol abuse, hepatitis C, portal hypertension, and non-alcoholic fatty liver, in addition to drug use and drug abuse, also contribute to the development of liver fibrosis [146].

In an in vivo study, Wang et al. [88] evaluated the effects of PS-modified nanostructured lipid carriers (mNLCs) containing CUR (Cur-mNLCs) to treat liver fibrosis induced in rats. The authors showed that the encapsulation of CUR in NLCs was efficient in enhancing the accumulation of the substance and its bioavailability in the tissues. These properties were increased by the addition of PS to the structures. The same logic applies to the delivery capability of NPs. Furthermore, Cur-mNLCs significantly reduced the fibrosis induced by carbon tetrachloride, upregulating the expression of hepatocyte growth factor (HGF) and MMP-2 in the liver, being the first important in the liver regeneration process.

Elzoheiry et al. [89] evaluated the therapeutic effects of CUR encapsulated in green silver NPs (AgNPs), coated or not with CH, in the treatment of liver fibrosis in vivo and in vitro. In the histological analysis of the hepatic tissue, it was possible to observe that the coated and non-coated NPs successfully potentiated the uptake of Cur by the hepatocytes; however, the increase was greater in the coated particles. Furthermore, analyses of liver enzymes levels demonstrated high levels of aspartate aminotransferase (AST) and alanine aminotransferase (ALT), as well as reduced levels of albumin, in the groups with induced liver fibrosis; only the group of animals treated with CUR/CH -coated AgNPs showed a significant reduction of the first two and albumin increase to parameters close to the negative control group. Furthermore, fibrosis-related genes were efficiently downregulated only by CUR/CH -coated AgNPs.

Chen et al. [90], in an in vitro and in vivo study, developed HA –polylactide NPs (HPNPs) in order to encapsulate CUR and potentiate its therapeutic effects in the treatment of liver fibrosis. The study was based on the idea that HA can be effective in increasing the uptake of NPs since its receptor, CD44, is excessively expressed in activated stellate cells (aHSCs) in the liver. *Hepatic stellate cells* (HSCs) isolated from rat livers were used as in vitro models, and Balb/c mice were used as in vivo models, in which liver fibrosis was induced from the intraperitoneal injection of thioacetamide. Histopathological analyses demonstrated that the CD44 receptor was positively regulated in liver tissue undergoing fibrosis. This fact allowed the specific uptake of CEHPNPs by aHSCs without the NPs presenting relevant interaction with healthy liver cells. Furthermore, the interaction of NPs without CUR with the tissue showed no relevant cytotoxicity, whereas, once loaded with CUR, the complex showed high cytotoxicity to aHSCs without, however, inducing the same apoptotic effect in healthy cells. Animals treated with CUR showed a reduction in serum levels of AST and ALT, as well as recovery of fibrotic tissue. Thus, by demonstrating similar therapeutic capabilities to the free form of CUR, however, reducing cytotoxicity, the use of CUR-encapsulated HA–polylactide NPs are a therapeutic alternative for the treatment of liver fibrosis.

In an in vitro and in vivo study, Atia et al. [91] evaluated the therapeutic effects of nanoCUR, when compared to free CUR, in the protection of hepatic tissue against acrylamide (AC)-induced fibrosis in Swiss albino mice. Treatment with Cur and nanoCUR showed similar results in reducing AST levels compared to the control group. However, nanoCUR was more effective in repressing ALT release). In addition, both compounds efficiently reduce the expression of pro-alpha1 chains of type I collagen (COL1A1) mRNA and thus mitigate liver fibrosis, with nanoCUR presenting better results than CUR. Furthermore, collagen deposition around hepatocytes, centrilobular vein, and sinusoid capillaries are also reduced with CUR and nanoCUR. Finally, the administration of CUR in the form of NPs showed better results than its free form, allowing the conclusion that this phytochemical is beneficial for the treatment of liver fibrosis but is also potentiated when in the form of NPs.

Adlia et al. [92] carried out an in vitro and in ovo study in which they aimed to measure the effectiveness of CUR in the treatment of liver fibrosis and, for this purpose, they combined CUR and gold (Au) with green chemistry to enhance the uptake of CUR by the hepatic tissue, as well as your activity. CURAuNPs proved to be a more potent antioxidant compared to free CUR, in addition to considerably reducing the amount of collagen observed in NIH/3T3 cells. Furthermore, in vitro studies allow us to state that CURAuNPs do not affect the viability of NIH/3T3 cells. Even more interestingly, concentrations below 300 µg/mL of the particles did not affect the development of embryos in ovo studies. In this way, the conjugation of CUR with gold NPs proved to be efficient, and in addition, the antioxidant activity was enhanced through the conjugation, while the toxicity of the phytochemical was reduced.

CUR-based nanomedicines can be used as potent anti-fibrotic adjuvants in fibrotic liver diseases. The authors of the included studies showed a reduction in AST, ALT, COL1A1 mRNA expression, hepatic fibrosis-related genes expression and hepatocytes, centrilobular vein and sinusoid capillaries collagen deposition, as well as increased hepatic albumin production, apoptosis of pro-inflammatory and pro-fibrotic cells, and HGF production levels.

### 3.9. CUR-Based Nanomedicines in COVID-19

**Figure 3:**
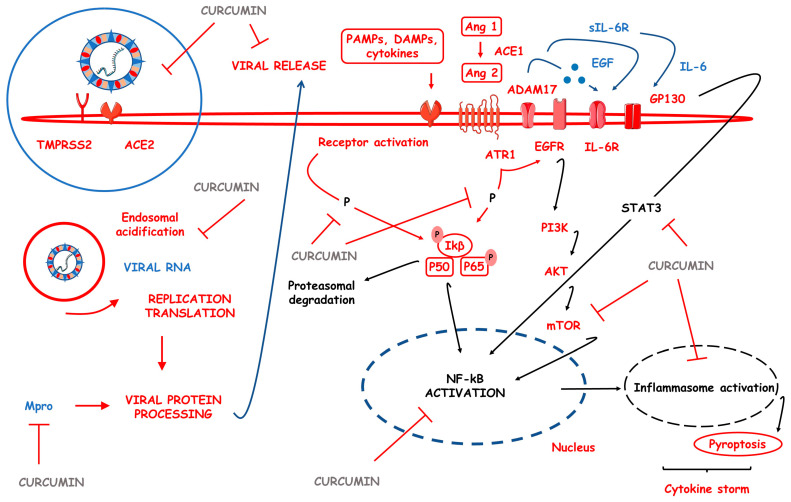
Effects of CUR not only on the inflammatory vias associated with COVID-19, but also with SARS-CoV-2 viral processing and release processes. CUR exerts principally anti-inflammatory and antioxidant effects against COVID-19, but also conducts immunomodulatory effects by controlling the expression of immunological genes. Regarding its use, CUR can also promote symptoms-relief during COVID-19 infection. ACE1, angiotensin-converting enzyme 1; ACE2, angiotensin-converting enzyme 2; ADAM17, disintegrin and metalloprotease 17; Ang 1, angiotensin 1; Ang 2, angiotensin 2; AKT, Alpha serine/threonine protein kinase; ATR1, angiotensin 1 receptor; DAMPs, damage-associated molecular patterns; EGF, epidermal growth factor; EGFR, epidermal growth factor receptor; GP130, glycoprotein 130; Ikβ, inhibitor of nuclear factor kappa β; IL, interleukin; IL-6R, interleukin 6 receptor; Mpro, main protease; mTOR, mammalian-target of rapamycin; NF-kB, nuclear factor kappa B; P50, the nuclear factor kappa B p50 heterodimer; P65, the nuclear factor kappa B p65 heterodimer; PAMPs, pathogen-associated molecular patterns; PI3K, phosphatidylinositol-3 kinase; sIL-6R, soluble form of the interleukin 6 receptor; TMPRSS2, transmembrane protease serine 2.

Coronavirus disease 2019 (COVID-19) is caused by the e severe acute respiratory syndrome coronavirus 2 (SARS-CoV-2). It has been long believed that the production of pro-inflammatory cytokines plays a critical role in the pathogenesis of this viral infection and promotes its gravity and high mortality rates in unvaccinated patients. Relevant evidence from severely ill patients suggested that a delayed release of cytokines and chemokines occurs in respiratory epithelial cells, macrophages, and dendritic cells at the early stage of COVID-19 infection. Later, immunological cells start to secrete many IFN factors, as well as different pro-inflammatory cytokines like IL-1β, IL-6, TNF-α, CCL2, CCL3, CCL4, and CCL5 [154,155]. Once an immunologic complication such as the cytokine storm occurs, only the antiviral treatment against SARS-CoV-2 becomes insufficient and should be used with appropriate anti-inflammatory treatment. Due to these reasons, synthetic anti-inflammatory drugs and corticosteroids gain space. However, these are high-cost and have serious adverse effects. In this scenario, natural phytochemicals that possess anti-inflammatory properties are important, and CUR is highlighted [156,157,158]. Figure 3 summarizes the effects of CUR not only on the inflammatory vias associated with COVID-19 but also with SARS-CoV-2 viral processing and release processes.

Hanafy & El-Kemary [99] studied the effects of silymarin/CUR-loaded albumin NPs coated by CH muco-inhalable delivery system as an anti-inflammatory against COVID-19 infection in vivo. The results showed that the mice levels of IL-6 and c reactive protein (CRP) were significantly reduced due to the nano treatment in comparison with the free treatment by not encapsulated or placebo capsules. However, only silymarin was effective in exerting antiviral therapy against COVID-19 and, therefore, ameliorated the histopathological parameters of the disease.

Asadirad et al. [100] conducted a placebo-controlled clinical trial to evaluate the efficacy of nanoCUR treatment against the typical inflammation and clinical manifestations of COVID-19 among male and female subjects. The intervention was conducted with 30 patients that received 240 mg/day nanoCUR for seven days orally and 30 controls that received a placebo. The results showed that nanoCUR effectively reduced the mRNA expression of IFN-γ, TNF-α, IL-1β, and IL-6 among the treated patients and ameliorated their clinical presentations. Therefore, the evidence demonstrated that nanoCUR could be effectively implicated as a complementary anti-inflammatory treatment agent against COVID-19 inflammatory complications.

Saber-Moghaddam et al. [97] studied nanoCUR as an efficacious formulation in the clinical management of hospitalized COVID-19 patients in an open-label, nonrandomized clinical trial. This study was conducted with 41 mild to moderate COVID-19 subjects. The treatment group received two capsules of sinaCUR soft gel containing 40 mg of CURoids as nanomicelles twice a day for two weeks orally. The results showed that the nanoCUR significantly improved patients’ recuperation velocity from fever, chills, myalgia, tachypnea, and cough. Additionally, nanoCUR effectively increased patients’ saturation of peripheral oxygen (SaO_2_) and decreased the duration of supplemented O_2_ and hospitalization. Therefore, oral nanoCUR could significantly improve the recovery time of mild to moderate COVID-19 subjects.

Hassaniazad et al. [98] conducted a triple-blind, placebo-controlled, randomized clinical trial to investigate the actions of nanoCUR capsules containing 40 mg of CUR as nanomicelles orally against mild to severe COVID-19. The subjects were divided into two groups, and the treatment received one capsule four times a day for two weeks. The results showed that nanoCUR effectively reduced IFN-γ significantly, IL-17, IL-4, and TGF-β serum levels, as well as reduced T-box transcription factor 21 (TBX21) and increased FoxP3 genetic expressions.

Ahmadi et al. [101], in a triple-blind, placebo-controlled, randomized clinical trial, studied the effects of nanoCUR treatment against mild to moderate COVID-19 infection. Treatment patients received two capsules twice daily for two weeks of sinaCUR soft gel containing 40 mg of CURoids as nanomicelles orally. The results showed that nanoCUR significantly increased patients’ recuperation velocity from chills, cough, and smell and taste disturbances and improved treated subjects’ lymphocyte counts.

Valizadeh et al. [96] conducted a double-blind, placebo-controlled, randomized clinical trial to investigate the actions of two capsules twice a day for two weeks of sinaCUR soft gel containing 40 mg of CURoids as nanomicelles orally against COVID-19. Results showed that the intervention effectively reduced serum patients’ IL-6, IL-18, IL-1β, and TNF-α. The authors highlighted that further trials are necessary to assess the effects of this treatment against clinical symptoms and signals of COVID-19.

Table 2 summarizes the effects of the above clinical studies in the Preferred Reporting Items for Systematic Reviews and Meta-Analyses (PRISMA) [159] style. All the included studies are from Iran [96,97,98,99,100,101]. A total of 241 COVID-19 patients were included, and many different nanoCUR formulations were used. The interventional range comprised periods from seven days to two weeks. The main results were increased recuperation velocity from COVID-19 symptoms and signals and reduced expression and release of pro-inflammatory cytokines and mediators. SinaCUR soft gel containing 40 mg of CURoids as nanomicelles orally was the most reproduced intervention as two capsules twice a day for two weeks. As shown by these results, it is possible to see the great potential of CUR-nanomedicines in this health condition.

## 4. Limitations and Future Perspectives

As commented before, CUR exerts potent anti-inflammatory, antioxidant, anti-amyloid, neuro-protective, and immune-modulating effects. However, its poor water solubility and low free-form absorption limit its use as a health-promoting agent. CUR-based nanomedicines have recently taken visibility to overcome these problematic characteristics and claimed greater treatment efficacy for CUR. This visibility can be justified as CUR became conjugated with not only surface stabilizers but also carriers with the highest cellular affinity to facilitate cellular uptake of CUR. Examples of high-affinity carriers include but are not limited to CH, PLGA, HAS, glycerol monooleate (GMO), polycaprolactone (PCL), and galactomannans [160]. In this scenario, delivery systems such as micelles, liposomes, nano-emulsions, micro-emulsions, phospholipid complexes, solid lipid NPs, biopolymer NPs, nanostructured lipid carriers, and microgels exhibit great promise [7]. These systems enhance CUR bioavailability by augmenting small intestine permeation, increasing the plasma half-life, and preventing possible degradation by the gastrointestinal tract microenvironment [161]. However, some limitations must be addressed. It should be noted that nano-sized structures exposed to large surface areas may lead to particle aggregation and, therefore, limited drug loading. In addition, there is a lack of studies in the scientific literature regarding the toxicity of NPs loaded with CUR in humans. Hence, further studies are necessary to evaluate whether CUR-loaded NPs could exhibit toxicity to humans, including neuroinflammation, DNA damage, allergic responses, and excitotoxicity [7,162,163].

CUR-nanoformulations are usually not tissue-specific. In other words, they are delivered to normal and diseased tissues along the body [5]. In studies that evaluated patients with COVID-19, which are the majority of clinical trials regarding nanoCUR that were included in the present manuscript, it seemed that the fact that nanoCUR arrives prominently in all organs did not preoccupy the investigators. Otherwise, COVID-19, as an infection-based condition, affected all human systems, even indirectly, due to the cytokine storm, and the synergistic effects of nanoCUR formulations among all tissues and organs were desired. In the near future, it should be noted that more consideration must be devoted to tissue-specificity in delivery methods based on nano drugs to counteract toxicity reactions and improve the drugs’ effectiveness.

## 5. Conclusions

The available studies showed that CUR-based nanoformulations could improve the bioavailability of this compound and can effectively be used as adjuvants in several inflammatory and immune-mediated diseases such as atheroma plaque formation, RA, dementia, AD, PD, HD, MS, IBD, psoriasis, COVID-19, and can be used as potent anti-fibrotic adjuvants in fibrotic liver disease. In addition, although nanomedicines are usually high cost, combination therapy of CUR in NPs can effectively decrease the daily dose of this bioactive compound. However, more studies are required to investigate CUR nanoformulations’ toxicity and confirm their efficacy in large numbers of human patients. Most of the published studies in the field are pre-clinical and correspond to in vitro and in vivo evidence. In addition, these were conclusive in pointing out the greater efficacy of CUR-based nanoformulations in improving CUR’s bioavailability. Most of all, the included studies compared interventions between free-CUR and nanoCUR agents in cell and animal models.

The prospects for CUR-nanomedicines envisioning leaving the laboratory for presence in the pharmacy are promising. They will significantly increase the effects of this polyphenol at an honest cost-benefit for patients. Besides that, the use of CUR-based nanomedicines is promising in medical theranostics. In this way, the individualized pharmacological need of the patient with inflammatory and immunomodulated diseases will be constantly and efficiently monitored.

## Figures and Tables

**Table 1:** Inflammatory and immunomodulated diseases and potential therapeutic effects of CUR-based nanomedicines against these diseases.

Diseases	Cur-Based Nanomedicines	Effects	References
Atherosclerosis	Polyvinylpyrrolidone and sodium dodecyl sulfate nanosuspensions, nanoparticles, and liposomes	↓TC, ↓LDL-c, ↓M1 macrophage polarization, and ↑M2 macrophage polarization, ↓progression of atheroma plaques, ↑inflammatory macrophages apoptosis, ↑atheroma plaques stability, ↓intraplaque micro vessels concentration, ↓MMP-2, ↓MMP-9, ↓pro-inflammatory cytokines production and release, ↓adhesion molecule expression, ↓monocyte migration into the intima layer of large to medium-sized arteries and ↓endothelial dysfunction	[36,37,38]
Rheumatoid arthritis	Nanoemulsions, solid lipid nanoparticles, nanoparticulate systems, and nanomicelles	↓NF-kB activation and signaling, ↓IL-1β, ↓IL-6, ↑IL-10, ↓TNF-α, ↑pain threshold, ↑joint mobility and stiffness, ↓inflammatory leukocytes recruitment, ↓ROS, ↓anti-CCP levels, ↓pannus formation, ↓bone destruction	[39,40,41,42]
Osteoarthritis	Nanoparticles, liposomes, CUR-loaded poly lactic-co-glycolic acid nanoparticles, extracellular vesicles and Poly(β -amino ester) amphiphilic polymers	↓IL-1β, ↓TNF-α, ↑expression of chondroprotective genes, ↓macrophages inflammatory differentiation in cartilages, ↓COX-2, ↓MMP-3, ↑cartilage glycosaminoglycan synthesis, ↓inflammatory cells migration to arthritis sites, ↓cartilage catabolic processes and ↓ROS	[43,44,45,46,47,48]
Alzheimer’s disease	Nanoliposomes, CUR-loaded PLGA nanoparticles, PLGA nanoparticles encapsulated in CUR, CUR liposomes conjugated with WGA and CL, CUR-loaded PLGA-PEG nanoparticles conjugated with B6 peptide, BSA-based CUR nanoparticles, selenium nanoparticles encapsulated PLGA nanospheres with CUR, solid lipid nanoparticles, highly-sensitive CUR-conjugated nanotheranostic platform and CUR lipid-core nanocapsules	↓Aβ aggregation, ↓amyloid fibril formation, ↑Aβ aggregates breakdown, ↑neurogenesis, ↑neuronal differentiation, ↑proliferation of endogenous neural stem cells, ↑β-catenin nuclear translocation, ↑GSK-3β phosphorylation, ↑expression of pro-neurogenic genes, ↑neuronal cells viability, ↑spatial learning, ↑memory capacity, ↓Tau phosphorylation, ↑microglial modulation, ↓brain inflammation (↓mRNA expression of TNF-α, IL-1β and IL-6, IFN-γ and NF-κB), ↓brain OS, ↓TG2	[49,50,51,52,53,54,55,56,57,58,59,60,61]
Parkinson’s disease	CUR-loaded lactoferrin n noparticles, CUR-loaded modified CPC nanoparticles, BSA-based nanoCUR, peptide-modified exosome chemical complex CURa/phenylboronic acid-poly(2 (dimethylamino) ethyl acr late) nanoparticle, and PLGA-lipid nanobubbles	↓α-synuclein expression, ↓brain OS, ↓TH, ↓Lewy body formation, ↓behavioral disturbances, ↓dopamine depletion, ↓neuronal cells death, ↑neuronal repair, ↑IL-10, ↓IL-2, ↓IL-17, and ↑dopamine transport to synaptic neurons	[62,63,64,65,66,67]
Multiple sclerosis	Dendrosomal CUR nanoparticles, CUR-HPPS, and simple nano CUR	↑Oligodendrogenesis, ↑remyelination, ↑neuronal myelin content, ↓astrocytes and microglia cells accumulation and actions, ↓microglial proliferation, ↓disease’s morbidity, ↓NF-kB activation and signaling, ↓adhesion and migration-related proteins, ↓peripheral Treg cell frequency and function, ↓TGF-β, IL-10 and FoxP3 expression levels, ↓inflammatory miR-145, miR-132, and miR-16 expression levels, ↓STAT1 activation and signaling, ↑STAT5 mRNA expression levels, ↓IL-1β, IL-6, CCL2, CCL5, IFN-γ, and TNF-α mRNA expression levels	[68,69,70,71,72]
Huntington’s disease	Solid lipid CUR nanoparticles	↓Striatum’s Complex II activity, ↑mitochondrial complexes activity, ↑cytochrome levels, ↓brain OS, ↑GSH, ↑SOD, ↓mitochondrial brain swelling, ↓brain lipid peroxidation, ↓protein carbonyls formation, ↓ROS production, ↑neuromotor coordination, and ↑Nrf2 activation and signaling	[73]
Inflammatory bowel diseases	PLGA-based CUR nanoparticles, hydrodynamic size CUR nanoparticles, chitosan capsule and unsaturated alginate resulting CUR nanoparticles, liposomes, nanocrystals, chondroitin sulfate CUR nanoparticles and porous CUR-loaded PLGA with PF127 nanoparticles	↓TNF-α, ↓IL-1β, ↓IL-6, ↓ROS, ↑HO-1, ↑IL-10, ↓NF-kB activation and signaling, ↓weight loss, ↓reduction in colon length, ↓increase in spleen size, ↓intestinal bleeding, ↓diarrhea, ↓levels of infiltrated neutrophils and macrophages and ↑maintenance of the original intestinal tissue architecture	[74,75,76,77,78,79,80,81,82]
Psoriasis	CUR-loaded HA-ES, CUR-loaded NLC, CUR-loaded Cur-CS/Alg nanoparticles, CUR nanuemulgel-based delivery system, and simple CUR nanoparticles	↓Inflammatory symptoms, ↓PASI, ↓TNF-α, ↓IL-17, ↓IL- 22, ↓IL-1β, ↓CCR6, ↓proliferation of psoriatic cells and ↓occurrence of psoriatic lesions	[83,84,85,86,87]
Liver fibrosis	mNLCs containing CUR, CUR encapsulated in AuNPs, CUR HPNPs, AgNPs, and simple nanoCUR	↓Hepatocytes, centrilobular vein and sinusoid capillaries collagen deposition and fibrosis, ↑HGF, ↓AST, ↓ALT, ↑albumin hepatic production, ↓hepatic fibrosis-related genes expression, ↑apoptosis of pro-inflammatory and pro-fibrotic cells and ↓COL1A1 mRNA expression	[88,89,90,91,92]
Epilepsy	CUR solid lipid NPs, CUR-loaded NPs, and CUR-loaded chitosan-alginate STPP NPs	↑Bcl-2 family progenitors activation, ↑P38 MAPK pathways activation, ↑behavioral performance, ↓neuronal apoptosis, ↓neuronal OS, ↑klotho levels, ↑EPO levels, ↓TNF-α mRNA levels, ↓microglia inflammatory activation and ↓memory deficits	[93,94,95]
COVID-19	Sinacurcumin soft gel containing 40 mg of curcuminoids as nanomicelles and NanoCUR capsules	↓IFN-γ, ↓TNF-α, ↓IL-6, ↓IL-17, ↓IL-4, ↓IL-1β, ↓TGF-β, ↓COVID-19 clinical aspects, ↑recuperation velocity from fever and chills, myalgia, tachypnea, cough and smell and taste disturbances, ↑SaO_2_, ↓duration of supplemented O_2_ and ↓duration of hospitalization, ↓TBX21 genetic expression and ↑FoxP3 genetic expression, ↑lymphocyte count	[96,97,98,99,100,101]

↑, increase; ↓, decrease; Aβ, beta amyloid; AgNPs, green silver nanoparticles; ALT, alanine transaminase; AST, aspartate transaminase; Bcl-2, B-cell lymphoma 2; BSA, bovine serum albumin; CCL2, chemokine (C-C motif) ligand 2; CCL5, chemokine (C-C motif) ligand 5; CCR6, chemokine receptor 6; CL, cardiolipin; COL1A1, pro-alpha1 chains of type I collagen; COVID-19, coronavirus disease 2019; COX-2, ciclooxigensase 2; CPC, polysorbate 80 cerassome; CUR, curcumin; Cur-CS/Alg, chitosan/alginate nanoparticles loaded with CUR; FoxP3, forkhead box protein 3; EPO, erythropoietin; GSH, glutathione; GSK-3β, glycogen synthase kinase 3; HA-ES, propylene glycol-based ethosomes modified with hyaluronic acid; HGF, hepatocyte growth factor; HO-1, heme oxygenase; HPNPs, hyaluronic acid–polylactide nanoparticles; HPPS, high-density lipoprotein-mimicking peptide-phospholipid scaffold; IFN, interferon; IL, interleukin; LDL-c, low-density lipoprotein cholesterol; MAPK, mitogen-activated protein kinases; MMP-2, matrix metalloproteinase 2; MMP-3, metalloproteinase 3; MMP-9, matrix metalloproteinase 9; mNLCs, PS-modified nanostructured lipid carriers; mRNA, messenger RNA [ribonucleic acid]; NF-kB, nuclear factor kappa b; NLC, nanostructured lipid carriers; Nrf2, nuclear factor erythroid 2–related factor 2; O_2_, oxygen; OS, oxidative stress; PASI, psoriasis area and severity index; PEG, polyethylene glycol; PF127, pluronic F127; PLGA, polylactic-coglycolic acid copolymer; ROS, reactive oxygen species; SaO_2_, saturation of peripheral oxygen; SOD, superoxide dismutase; STAT1, signal transducer and activator of transcription 1; STAT5, signal transducer and activator of transcription 5; STPP, sodium tripolyphosphate; TBX21, T-box transcription factor 21, TC, total cholesterol; TG2, tissue transglutaminase isoform; TGF-β, transforming growth factor beta; TH, tyrosine hydroxylase; TNF-α, tumor factor necrosis alfa; WGA, wheat germ agglutinin.

**Table 2:** Information of studies regarding the use of CUR-based nanomedicines in COVID-19 patients summarized following PRISMA [159] style.

**Reference**	**Study**	**Population**	**Intervention**	**Duration**	**Outcomes**
[100]	Placebo-controlled clinical trial (Iran)	60 COVID-19 patients randomly divided equally into nanoCUR (56 ± 14.02 y, 24♂ and 6♀) and placebo (50.2 ± 12.01 y, 24♂ and 6♀) groups	240 mg/day of nanoCUR orally	7 days	↓IFN-γ mRNA, ↓TNF-α mRNA, ↓IL-6 mRNA, ↓IL-1β and ↓COVID-19 clinical aspects
[97]	Open-label, nonrandomized clinical trial (Iran)	41 mild to moderate COVID-19 patients allocated into nanoCUR (*n* = 21, 53.48 ± 12.21 y, 5♂ and 16♀) and placebo (*n* = 20, 58.45 ± 17.71 y, 9♂ and 11♀) groups	SinaCUR soft gel containing 40 mg of CURoids as nanomicelles orally	Two capsules twice a day/2 weeks	↑Recuperation velocity from fever and chills, myalgia, tachypnea and cough, ↑SaO_2_, ↓duration of supplemented O_2,_ and ↓duration of hospitalization
[98]	Triple-blind, placebo-controlled, randomized clinical trial (Iran)	40 mild to severe COVID-19 patients allocated equally into nanoCUR (48.7 ± 10.8 y, 10♂ and 10♀) and placebo (48.3 ± 11 y, 12♂ and 8♀) groups	NanoCUR capsules containing 40 mg of CUR as nanomicelles orally	One capsule four times a day for 2 weeks	↓IFN-γ, ↓IL-17, ↓IL-4, ↓TGF-β, ↓TBX21 genetic expression and ↑FoxP3 genetic expression
[101]	Triple-blind, placebo-controlled, randomized clinical trial (Iran)	60 mild to moderate COVID-19 patients equally allocated into nanoCUR (41.33 ± 12.04 y, 20♂ and 10♀) and placebo (44.97 ± 11 y, 15♂ and 15♀) groups	SinaCUR soft gel containing 40 mg of CURoids as nanomicelles orally	Two capsules twice a day/2 weeks	↑Recuperation velocity from chills, cough and smell and taste disturbances and ↑lymphocyte count
[96]	Double-blind, placebo-controlled, randomized clinical trial (Iran)	40 COVID-19 patients equally divided into nanoCUR (53.3 ± 8.4 y, 15♂ and 5♀) and placebo (51.4 ± 7.9 y, 16♂ and 4♀) groups	SinaCUR soft gel containing 40 mg of CURoids as nanomicelles orally	Two capsules twice a day/2 weeks	↓IL-6, ↓IL-18, ↓IL-1β and ↓TNF-α

↑, increase; ↓, decrease; ♂, male; ♀, female; COVID-19, coronavirus disease 2019; CUR, curcumin; FoxP3, forkhead box protein 3; IFN-γ, interferon-gamma; IL, interleukin; O_2_, oxygen; SaO_2_, saturation of peripheral oxygen; TBX21, T-box transcription factor 21; TGF-β, transforming growth factor beta; TNF-α, tumor factor necrosis alfa; y, years.

## Data Availability

Not applicable.
